# High-resolution pediatric age–specific ^18^F-FDG PET template: a pilot study in epileptogenic focus localization

**DOI:** 10.1007/s00259-021-05611-w

**Published:** 2021-11-08

**Authors:** Teng Zhang, Yuting Li, Shuilin Zhao, Yuanfan Xu, Xiaohui Zhang, Shuang Wu, Xiaofeng Dou, Congcong Yu, Jianhua Feng, Yao Ding, Junming Zhu, Zexin Chen, Hong Zhang, Mei Tian

**Affiliations:** 1 Department of Nuclear Medicine and Medical PET Center, The Second Hospital of Zhejiang University School of Medicine, 88 Jiefang Road, Hangzhou, 310009 Zhejiang China; 2 Institute of Nuclear Medicine and Molecular Imaging of Zhejiang University, Hangzhou, China; 3 Key Laboratory for Biomedical Engineering of Ministry of Education, Zhejiang University, Hangzhou, China; 4 Hangzhou Universal Medical Imaging Diagnostic Center, Hangzhou, China; 5 Department of Pediatrics, The Second Hospital of Zhejiang University School of Medicine, Hangzhou, China; 6 Department of Neurology, Epilepsy Center, The Second Hospital of Zhejiang University School of Medicine, Hangzhou, China; 7 Department of Neurosurgery, The Second Hospital of Zhejiang University School of Medicine, Hangzhou, China; 8 Center of Clinical Epidemiology & Biostatistics, The Second Hospital of Zhejiang University School of Medicine, Hangzhou, China; 9 The College of Biomedical Engineering and Instrument Science, Zhejiang University, Hangzhou, China

**Keywords:** Epilepsy, Positron emission tomography (PET), Template, Pediatric age–specific

## Abstract

**Background:**

PET imaging has been widely used in diagnosis of neurological disorders; however, its application to pediatric population is limited due to lacking pediatric age–specific PET template. This study aims to develop a pediatric age–specific PET template (PAPT) and conduct a pilot study of epileptogenic focus localization in pediatric epilepsy.

**Methods:**

We recruited 130 pediatric patients with epilepsy and 102 age-matched controls who underwent ^18^F-FDG PET examination. High-resolution PAPT was developed by an iterative nonlinear registration-averaging optimization approach for two age ranges: 6–10 years (*n* = 17) and 11–18 years (*n* = 50), respectively. Spatial normalization to the PAPT was evaluated by registration similarities of 35 validation controls, followed by estimation of potential registration biases. In a pilot study, epileptogenic focus was localized by PAPT-based voxel-wise statistical analysis, compared with multi-disciplinary team (MDT) diagnosis, and validated by follow-up of patients who underwent epilepsy surgery. Furthermore, epileptogenic focus localization results were compared among three templates (PAPT, conventional adult template, and a previously reported pediatric linear template).

**Results:**

Spatial normalization to the PAPT significantly improved registration similarities (*P* < 0.001), and nearly eliminated regions of potential biases (< 2% of whole brain volume). The PAPT-based epileptogenic focus localization achieved a substantial agreement with MDT diagnosis (Kappa = 0.757), significantly outperforming localization based on the adult template (Kappa = 0.496) and linear template (Kappa = 0.569) (*P* < 0.05). The PAPT-based localization achieved the highest detection rate (89.2%) and accuracy (80.0%). In postsurgical seizure-free patients (*n* = 40), the PAPT-based localization also achieved a substantial agreement with resection areas (Kappa = 0.743), and the highest detection rate (95%) and accuracy (80.0%).

**Conclusion:**

The PAPT can significantly improve spatial normalization and epileptogenic focus localization in pediatric epilepsy. Future pediatric neuroimaging studies can also benefit from the unbiased spatial normalization by PAPT.

Trial registration.

NCT04725162: https://clinicaltrials.gov/ct2/show/NCT04725162

**Supplementary Information:**

The online version contains supplementary material available at 10.1007/s00259-021-05611-w.

## Introduction

Positron emission tomography (PET) imaging provides an in-depth evaluation of physiological and pathological processes of in vivo brains and has been widely used in the investigation of neurological disorders [1, 2]. The current research and diagnosis of PET imaging usually depend on spatial normalization to the standard adult brain template for tissue segmentation, voxel-wise statistics, diagnosis, and classification of diseases [3–5]. However, the standard adult template is not adequate for investigation of pediatric PET images due to the large morphological and metabolic differences between adult and pediatric brains, which could lead to anatomical misalignment, and in consequence, loss of diagnostic efficiency [6, 7]. Moreover, the most commonly used PET template is that provided by statistical parametric mapping (SPM), which was built by averaging and smoothing ^15^O-H_2_O PET images from 12 healthy controls. Because of the large difference between intensity distribution of ^15^O-H_2_O and ^18^F-FDG PET images, the SPM template could lead to inevitable misalignment and reduced sensitivities in ^18^F-FDG PET diagnosis [8, 9].

As we know, many magnetic resonance imaging (MRI) studies developed pediatric age–specific MRI templates, which can significantly improve spatial normalization, morphological analysis, and clinical diagnosis [10–12]. However, to the best of our knowledge, almost no pediatric PET brain template has been released for diagnosis of pediatric neurological diseases. Only one previous study reported creation of an in-house pediatric ^18^F-FDG PET template based on linear registration between PET images and pediatric MRI template [13]. However, the linear registration transformed whole image in the same manner and thus could blur anatomical details in cortical regions [12]. In addition, age ranges were not well matched between MRI template and PET data in the previous study, which may introduce systematic biases to spatial normalization due to rapid brain development during childhood [11, 12].

Therefore, in this study, we created pediatric age–specific ^18^F-FDG PET template (PAPT) based on a nonlinear optimization method to overcome the above issues. The efficiency of our template was evaluated by epileptogenic focus localization in pediatric patients with epilepsy. The localization results were further compared with clinical diagnosis and validated by follow-up of patients who underwent epilepsy surgery.

## Materials and methods

### Subjects

We retrospectively reviewed a dataset of 591 pediatric patients with epilepsy (265 girls and 326 boys) who underwent ^18^F-FDG PET/computed tomography (CT) imaging at our hospital from October 2013 to December 2020. The inclusion criteria included (a) age between 6 and 18 years; (b) clinical diagnosis as focal epilepsy; (c) detailed seizure semiology evaluation, MRI, EEG, and interictal ^18^F-FDG PET/CT examination; (d) last seizure occurring more than 48 h before PET/CT examination. The exclusion criteria included (a) any history of central nervous system disease other than epilepsy; (b) diagnosis as multifocal epilepsy, epilepsy syndrome, or epileptic encephalopathy; (c) poor image quality due to head movement; (d) uncertain epileptogenic focus. At last, a total of 130 patients were included for the following analysis (59 girls and 71 boys, mean age = 12.46 years).

The pseudo-controls included 102 children with extracranial tumors (age range: 6–18 years). All controls had no history of any neuropsychiatric disorders and never received any treatment before PET imaging which could affect brain metabolism, such as radiotherapy and chemotherapy. Two experienced physicians carefully reviewed imaging data of pseudo-controls, and excluded subjects with any brain structural or metabolic abnormalities. Sixty-seven controls (21 girls and 46 boys, mean age = 13.08 years) underwent PET/CT imaging at our hospital, and 35 controls (14 girls and 21 boys, mean age = 11.93 years) underwent PET/MR imaging at Hangzhou Universal Medical Imaging Diagnostic Center. This retrospective study was approved by the Human Subject Research Ethics Committee of the Second Hospital of Zhejiang University School of Medicine, and requirement of informed consent was waived (approval no. 2021–0190).

### ^*18*^*F-FDG PET imaging*

^18^F-FDG PET/CT images were acquired by a PET/CT scanner (Biograph mCT; Siemens Medical Solution) and ^18^F-FDG PET/MR images were acquired by a PET/MR scanner (Signa PET/MR; GE Healthcare). All subjects fasted for at least 6 h such that the pre-scan plasma glucose level < 120 mg/dL [14]. Subjects were injected with a standard dose of ^18^F-FDG (3.7 MBq/Kg), rested in a dark quiet environment 40-min for glucose uptake, and underwent a 5-min three-dimensional brain scan. The PET images were reconstructed into a 400 × 400 × 400 matrix with a voxel size of 1.0 mm × 1.0 mm × 0.75 mm and were attenuation corrected by vendor-provided software.

### Development of pediatric age–specific PET templates

**Fig. 1:**
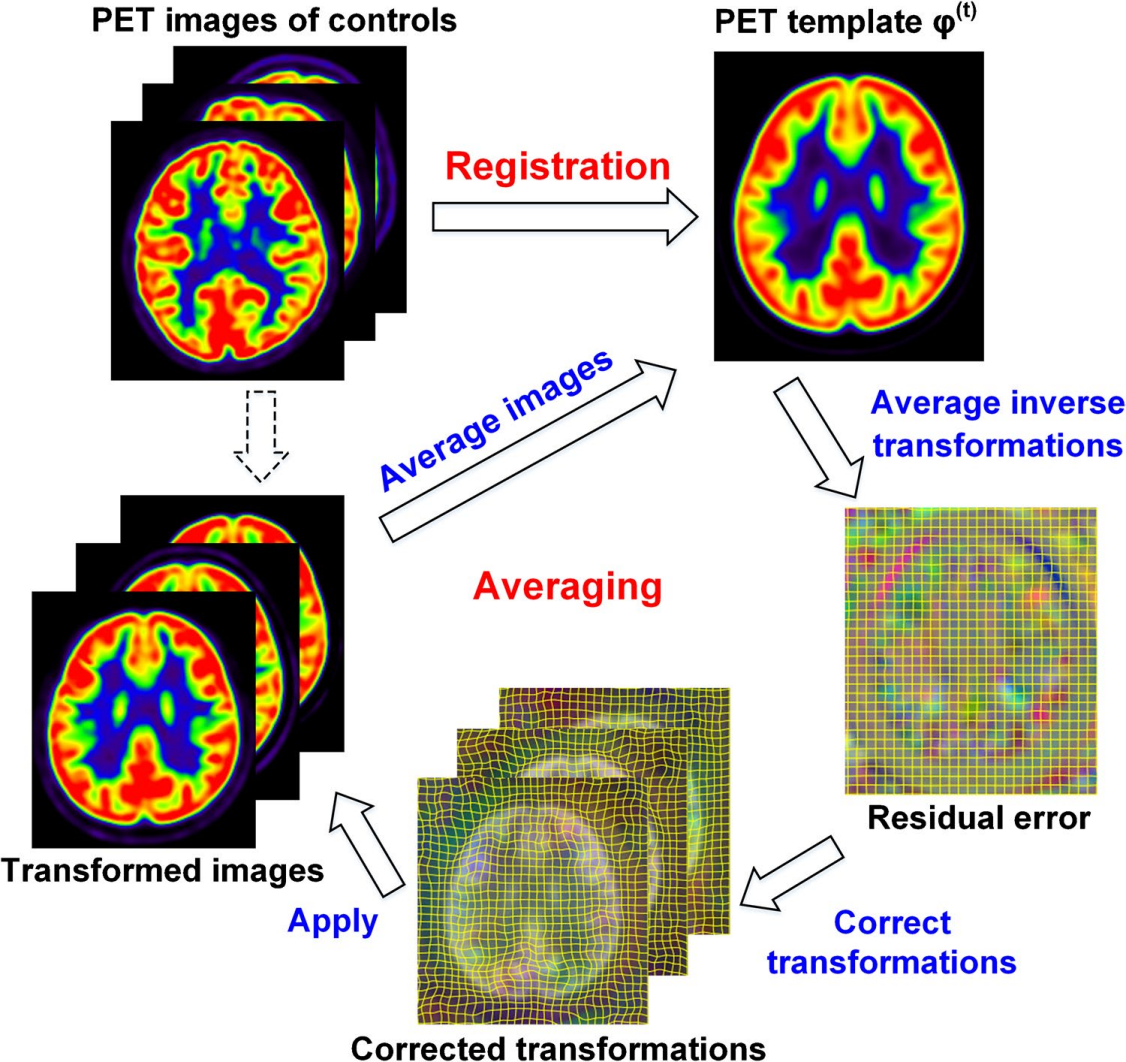
Flowchart of iterative registration-averaging optimization. This optimization approach iterated between registration and averaging steps to create unbiased brain template

The pediatric age–specific PET template (PAPT) was developed from PET/CT controls. PET images were first intensity normalized by using whole brain average standardized uptake value (SUV) as the reference. Then, an iterative registration-averaging optimization approach was utilized to develop PAPT (Fig. 1) [12]. Given $n$ PET images $\left({J}_{1},{J}_{2},\cdots {J}_{n}\right)$, let ${\theta }_{i}$ denote transformation from ${J}_{i}$ to an approximated template image ${\varphi }$. The PAPT $\phi$ is the template that minimizes total metabolic differences between spatially normalized image ${\theta }_{i}\left({J}_{i}\right)$ and itself:

$$\phi =\underset{\varphi }{\mathrm{argmin}}\sum_{i=1}^{n}{\Vert \varphi -{\theta }_{i}\left({J}_{i}\right)\Vert }^{2}$$

To best represent anatomical structures within the population, the PAPT also follows a morphological constraint that the minimal deformation is required to transform individual PET images to PAPT space:

$$\phi =\underset{\varphi }{\mathrm{argmin}}\sum_{i=1}^{n}{\Vert {\theta }_{i}\Vert }^{2}$$

The iterative optimization procedure satisfied the two constraints simultaneously by repeating registration and averaging steps. At iteration *t*, the registration step spatially normalized all PET images to an approximated template image ${\varphi }^{\left(t\right)}$. The averaging steps included:

1. Average non-rigid components in the inverse transformation of ${\theta }_{i}^{\left(t\right)}$ as residual error ${R}^{\left(t\right)}$. The residual error characterizes unwanted deformations of spatial normalization.
2. Correct transformations by composition with residual error: ${\widehat{\theta }}_{i}^{\left(t\right)}={R}^{\left(t\right)}\bullet {\theta }_{i}^{\left(t\right)}$. In this way, unwanted deformations can be removed to satisfy the morphological constraint.
3. Transform PET images by corrected transformations, and average transformed images as the approximated template image in the next iteration: ${\varphi }^{\left(t+1\right)}=\sum {\widehat{\theta }}_{i}^{\left(t\right)}\left({J}_{i}\right)/n$. This step satisfies the metabolic constraint.

The development procedure stopped when the mean squared difference between two consecutive template images was less than convergence level (0.0001). In concern of computing speed, a coarse-to-fine creation approach was adopted by using affine registration, 2-mm symmetric diffeomorphic normalization (SyN) nonlinear registration and 1-mm SyN sequentially [15]. To correct transformations, matrix multiplication was used for affine registration, and field composition for SyN registration: ${D}_{1}\bullet {D}_{2}\left(v\right)={D}_{1}\left(v\right)+{D}_{2}\left(v+{D}_{1}\left(v\right)\right)$, where ${D}_{1}$ and ${D}_{2}$ were two deformation fields, $v$ denoted voxel coordinate. After development, brain regions of PAPT were partitioned according to the Desikan-Killiany atlas [16], which could provide anatomical information for future studies (Supplementary Materials S1). The template development process was implemented by an in-house software based on Insight Toolkits library (http://www.itk.org/).

### Epilepsy focus localization

All ^18^F-FDG PET images of patients were spatially normalized to the PAPT by affine and SyN registration with following parameters: multiresolution level = 3, maximum iterations = 100 × 100 × 100, learning rate = 0.25, convergence threshold = 10^−6^, convergence window size = 10, variance for total field = 0.5, and variance for update field = 3. Normalized correlation was used for affine registration, and neighborhood correlation coefficient (NCC) was used for SyN registration. SyN registration was used since it achieved the best performance in comparison to 14 nonlinear registration algorithms by a previous study [17], and it could improve spatial normalization compared with SPM registration (Supplementary S2).

The spatially normalized images were Gaussian smoothed by a 4-mm full-width half maximum (FWHM) because of small PET voxel size in this study. Supplementary S3 compares different smoothing kernels and shows that FWHM = 4 mm was appropriate for PET data in this study. SUV ratios (SUVRs) were calculated by using average SUV in cerebellum gray matter (GM) as the reference:

$$\mathrm{SUVR}=\mathrm{SUV}/\mathrm{average}\left({\mathrm{SUV}}_{\mathrm{Cerebellum GM}}\right)$$

Metabolic abnormalities were determined by voxel-wise statistical analysis using SPM software (https://www.fil.ion.ucl.ac.uk/spm). Single-subject PET image was compared with age-specific control data (6–10 years: *n* = 17; 11–18 years: *n* = 50). No covariate was used in SPM statistical analysis. Clusters of contiguous voxels (*P* < 0.01 uncorrected and cluster size > 100) were extracted (selection of the thresholds as described in Supplementary S4). The most significant cluster, i.e., cluster containing the maximal absolute peak-*t*-value, was considered the epileptogenic focus. Lobe containing the peak-*t*-value was identified according to Desikan-Killiany atlas in PAPT space.

### Comparison of brain templates

The PAPT was compared with a previously reported pediatric linear template and SPM built-in adult template. According to a previous study [13], the pediatric linear template was created by linear registration with an existing MRI template. PET images of PET/MR controls were spatially normalized to the three templates by affine and SyN registration. Then, registration similarities of the spatial normalization were quantified by subject-template and inter-subject neighborhood cross-correlation (NCC) and compared among three templates by repeated-measures analysis of variance (ANOVA) with post hoc. Thereafter, logarithm scaling parameters (log-scaling) were decomposed from affine transformation matrices and tested by a one-sample *t*-test. Positive and negative log-scaling indicated global stretching and shrinkage, respectively. At last, registration bias was estimated by logarithm Jacobian determinant ($\mathrm{log}\left|{\varvec{J}}\right|$) of deformation fields. The positive and negative $\mathrm{log}\left|{\varvec{J}}\right|$ indicated registration biases of local expansion and shrinkage, respectively [12]. Voxel-wise one-sample *t*-test was performed to test whether $\mathrm{log}\left|{\varvec{J}}\right|$ equaled to 0, followed by a false discovery rate (FDR) of 5% to account for multiple comparisons.

### Evaluation of epileptogenic focus localization

Epileptogenic focus was diagnosed by multi-disciplinary team (MDT) according to seizure semiology, EEG, MRI, and ^18^F-FDG PET/CT findings for no-surgery patients. The ^18^F-FDG PET images were visually assessed in MDT diagnosis. Resection areas on postsurgical structural images were used to evaluate focus localization of patients who underwent epilepsy surgery. Focus lateralization (left or right) and localization lobes (frontal, temporal, parietal, or occipital lobe) were determined.

Localization of PAPT-based analysis was compared with the MDT diagnosis by Kappa test and furthermore investigated in patients whose foci were missed by routine visual assessment. The Kappa statistics indicated fair (0.21–0.40), moderate (0.41–0.60), moderate (0.61–0.80), and almost perfect (0.81–0.99) agreements. Localization accuracy was defined as the proportion of patients whose most significant cluster was consistent with MDT diagnosis. Detection rate was defined as the proportion of patient whose focus was identified. Focus localization of analyses based on three templates (the PAPT, linear template, and adult template) was compared by McNemar test. At last, cluster size and confounding cluster (i.e., the most significant cluster inconsistent with MDT diagnosis) were investigated.

### Validation in postsurgical patients

Focus localization results were validated in patients who underwent epilepsy surgery. According to the Engel classification criteria, postsurgical patients were identified as seizure-free (class I) or non-seizure-free (class II–IV) [18]. Epileptogenic foci of the seizure-free patients could be successfully resected by epilepsy surgery; therefore, resection regions on their postsurgical MRI were used as gold standard. Postsurgical MRI images were coregistered with corresponding presurgical PET images by rigid registration. Localization was considered to be correct when the most significant cluster fell within resection area. Kappa test was performed to evaluate agreement between localization and gold standard. Accuracy and detection rate were estimated, followed by McNemar test to compare localization based on different templates.

## Results

### Pediatric age–specific PET templates

**Fig. 2:**
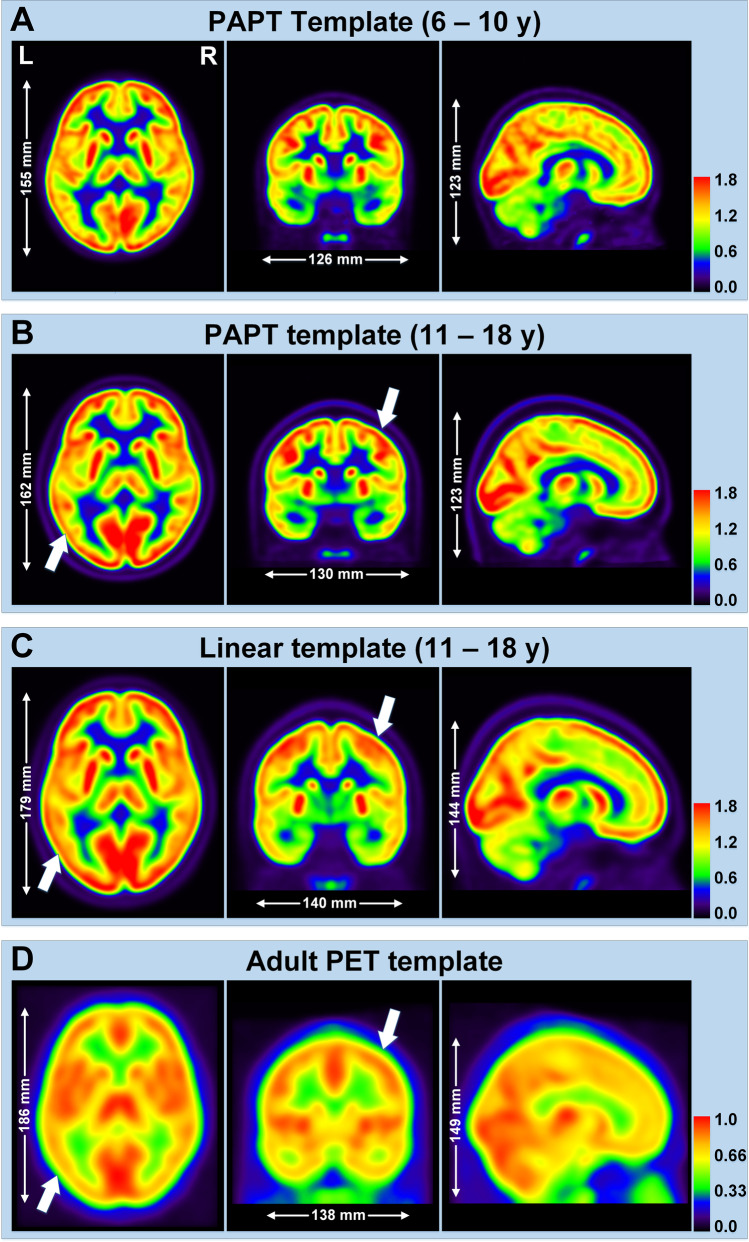
Comparison of PET templates. **A** 6–10 years PAPT; **B** 11–18 years PAPT; **C** pediatric linear template; **D** adult PET template. Arrows point blurred cortical structures in linear and adult templates

The 6–10-year PAPT was developed from 17 PET/CT controls (4 girls and 13 boys, mean age = 8.31 years) and the 11–18-year PAPT from 50 PET/CT controls (17 girls and 33 boys, mean age = 14.69 years) (Fig. 2A, B). Brain parcellation of PAPT is shown in Supplementary Fig. 1, and convergence process in Supplementary Fig. 2. The 6–10-year PAPT had smaller length (155 mm) and width (126 mm) than the 11–18-year PAPT (length = 162 mm, width = 130 mm) and both had the same height of 123 mm. Compared with PAPT, linear and adult templates had larger brain sizes and more blurred structural details (Fig. 2C, D).

### Comparison of brain templates

**Fig. 3:**
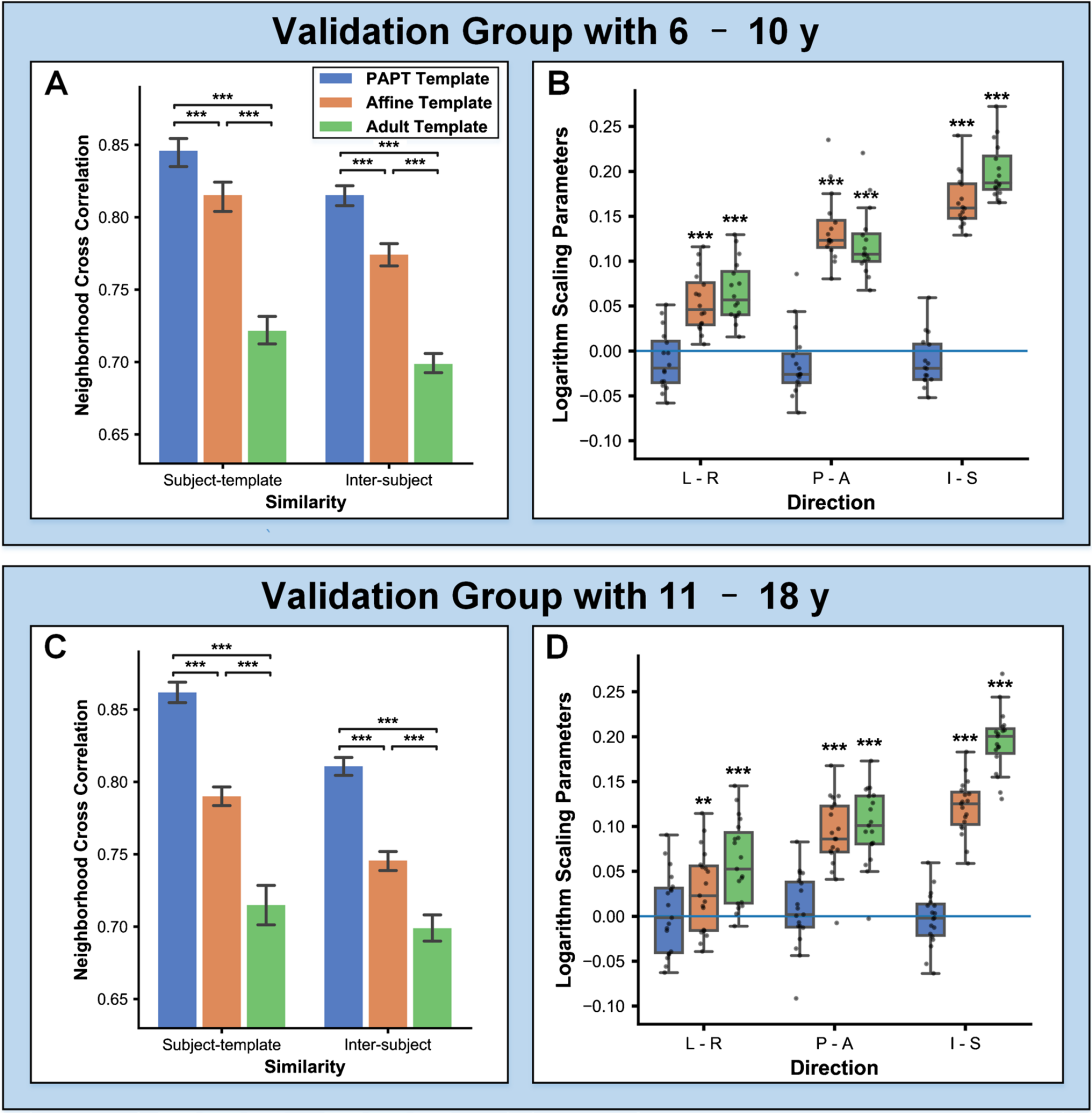
Comparison of spatial normalization. **A**, **B** Registration similarities and global transformation for the 6–10 years group, respectively; **C**, **D** 11–18 years group

Controls who underwent PET/MR examination were included to validate efficiency of spatial normalization. These PET/MR controls were divided into 6–10 years group (7 girls and 9 boys, mean age = 8.12 years) and 11–18 years group (7 girls and 12 boys, mean age = 14.16 years) for template comparison. For the 6–10 years group, spatial normalization to the PAPT had significantly increased registration similarities (subject-template NCC: 0.846 vs. 0.816 and 0.722; inter-subject NCC: 0.816 vs. 0.774 and 0.699) compared with those to linear and adult templates (*P* < 0.001) (Fig. 3A). For the 11–18 years group, spatial normalization to the PAPT also had significantly increased subject-template (0.862 vs. 0.790 and 0.715) and inter-subject NCCs (0.811 vs. 0.746 and 0.699) (*P* < 0.001), as shown in Fig. 3C.

**Fig. 4:**
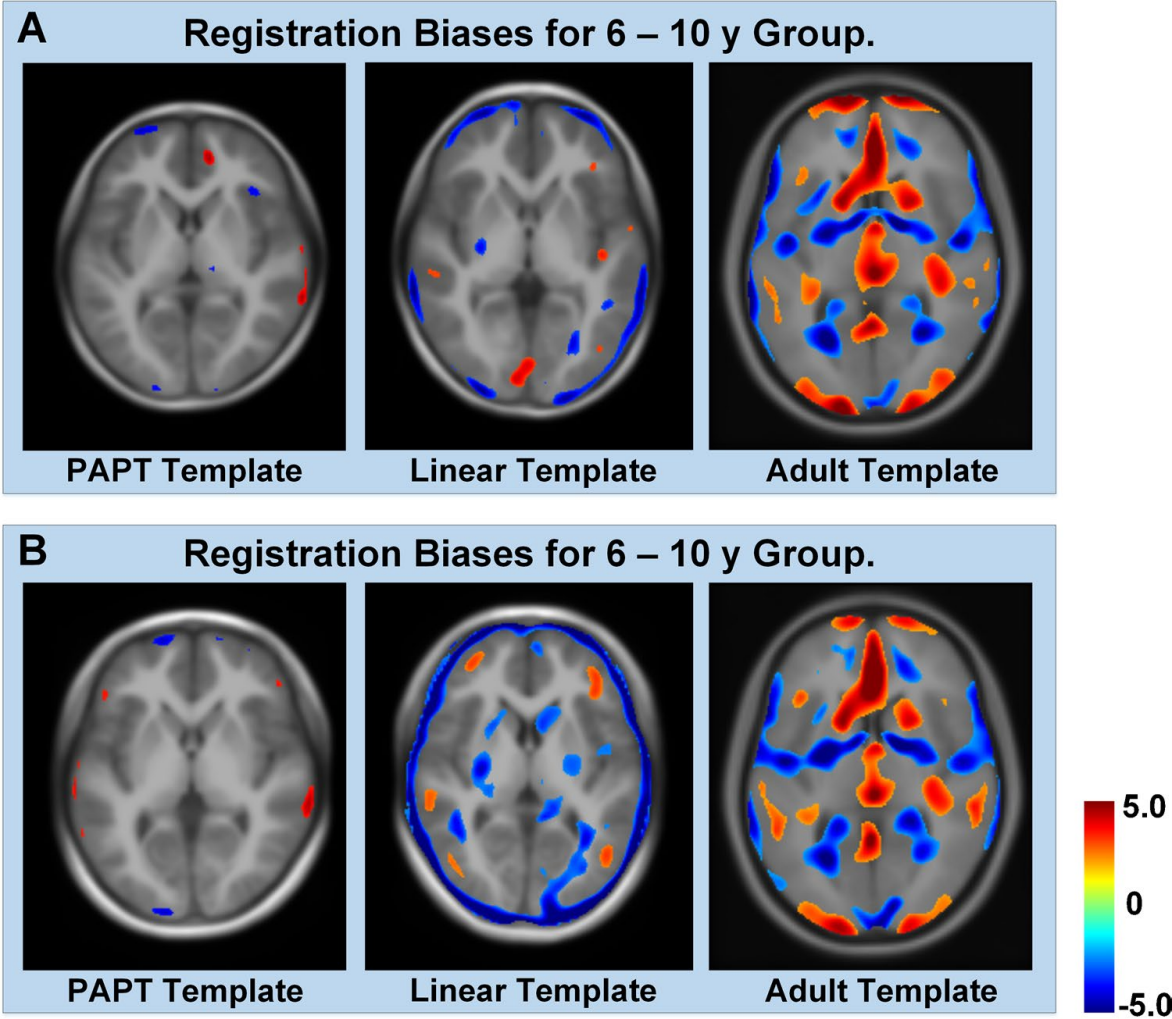
Regions of potential registration bias in spatial normalization. **A** 6–10 years group; **B** 11–18 years group

Figure 3B and D demonstrate that spatial normalization to the linear template and adult template required significant global stretching along with three directions (log-scaling = 0.028–0.200, *P* = 0.000–0.015), while no unwanted global stretching or shrinkage was needed when normalizing to the PAPT (log-scaling =  − 0.013–0.006, *P* = 0.126–0.754). The detailed global transformation parameters are shown in Supplementary Table 1. As demonstrated in Fig. 4, regions of potential registration biases were about 1.54% and 1.26% of whole brain volume when normalizing to our PAPT for the 6–10 years and 11–18 years groups, respectively. Meanwhile, the regions of registration biases were 5.68% and 18.04% on the linear template and 35.62% and 40.58% on the adult template, mainly located on cortical regions.

### Epileptogenic focus localization results

Pediatric patients with epilepsy were divided into 6–10 years group (20 girls and 28 boys, mean age = 8.88 years) and 11–18 years group (39 girls and 43 boys, mean age = 14.56 years). The PAPT-based analysis achieved a substantial agreement with MDT diagnoses in both groups (Kappa = 0.710 and 0.771), while analyses based on linear template (Kappa = 0.485 and 0.595) and adult template (Kappa = 0.461 and 0.491) achieved only moderate agreements. Among three analyses, the PAPT-based analysis achieved the highest detection rate (89.2%) and accuracy (80.0%) (Table 1).

**Table 1:** Epileptogenic focus localization results

	Kappa (95% CI)	Detection rate	Accuracy
6–10 years group (*n* = 48)			
PAPT template	0.710 (0.567–0.853)	83.3%	77.1%
Linear template	0.485 (0.316–0.654)	75.0%	60.4%
Adult template	0.461 (0.298–0.623)	75.0%	58.3%
11–18 years group (*n* = 82)			
PAPT template	0.771 (0.669–0.872)	92.7%	81.7%
Linear template	0.595 (0.479–0.711)	85.4%	67.1%
Adult template	0.491 (0.368–0.614)	84.1%	58.5%
All patients (*n* = 130)			
PAPT template	0.757 (0.675–0.839)	89.2%	80.0%
Linear template	0.569 (0.475–0.663)	81.5%	64.6%
Adult template	0.496 (0.400–0.592)	80.8%	58.5%

*CI*, confidence interval; *PAPT*, pediatric age–specific PET template

**Fig. 5:**
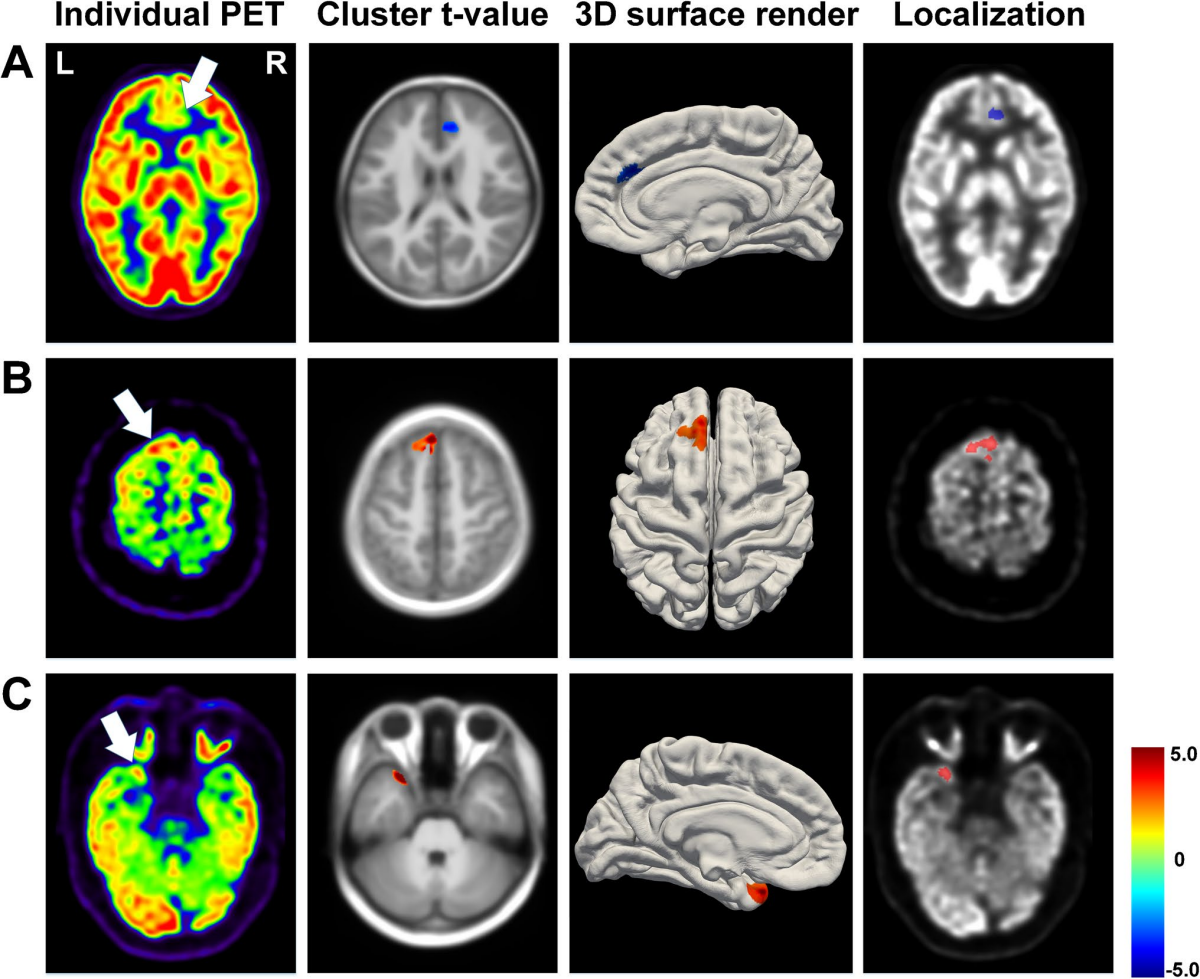
Localization results of patients whose foci were missed by routine visual assessment. **A** A 9-year-old boy: right frontal lobe hypo-metabolism, peak-*t* =  − 4.04, cluster size = 963; **B** 18-year-old boy: left frontal lobe hyper-metabolism, peak-*t* = 7.37, cluster size = 1387; **C** 15-year-old boy: left temporal lobe hyper-metabolism, peak-*t* = 8.16, cluster size = 452 (*P* < 0.01, cluster size > 100)

Routine visual assessment missed epileptogenic foci in 8 patients with temporal lobe epilepsy (TLE) and 37 with extra-TLE. Twelve of these patients showed hyper-metabolism, and six were visually diagnosed as contralateral hypo-metabolism. The PAPT-based analysis identified 82.2% (8 TLE and 29 extra-TLE) of these patients, while the linear and adult template-based analyses could only identify 71.1% and 64.5% of these patients, respectively. In total, 19 patients showed interictal hyper-metabolism, and all could be detected by PAPT-based analysis. Figure 5 illustrates localization results of 3 representative patients (A–B: visually negative patients with hypo- and hyper-metabolism, respectively; C: a patient who was visually diagnosed as contralateral hypo-metabolism). Furthermore, visual assessment could detect foci in 7 of 14 patients with negative findings in PAPT-based analysis.

In both age groups, the PAPT-based analysis significantly outperformed analyses based on linear template (*P* = 0.035 and 0.004) and adult template (*P* = 0.006 and *P* < 0.001). Linear template had similar performance with adult template in the 6–10 years group (*P* = 1.0), and marginally better performance in the 11–18 years group (*P* = 0.065). In the 116 patients with detected foci, 94 (81.0%) had the largest cluster sizes. In the 12 patients with confounding clusters, 9 foci clusters had the largest sizes.

### Validation in postsurgical patients

Fifty patients underwent epilepsy surgery with a mean follow-up of 2.26 years. According to the Engel classification criteria, 14 patients with 6–10 years (6 girls and 8 boys, mean age = 8.72 years) and 26 patients with 11–18 years (13 girls and 13 boys, mean age = 14.61 years) were postsurgical seizure-free. As shown in Table 2, localization based on PAPT achieved a substantial agreement with the resection areas (Kappa = 0.743), significantly outperforming those based on pediatric linear template (Kappa = 0.536, *P* = 0.039) and adult template (Kappa = 0.469, *P* = 0.004). In addition, localization based on linear template and adult template showed no significant difference (*P* = 0.625).

**Table 2:** Epileptogenic focus localization results for postsurgical seizure-free patients

	Kappa (95% CI)	Detection rate	Accuracy
6–10 years seizure-free patients (*n* = 14)			
PAPT template	0.800 (0.553–1.000)	100.0%	85.7%
Linear template	0.468 (0.158–0.778)	92.9%	57.1%
Adult template	0.436 (0.115–0.757)	92.9%	57.1%
11–18 years seizure-free patients (*n* = 26)			
PAPT template	0.685 (0.475–0.895)	92.3%	76.9%
Linear template	0.536 (0.322–0.750)	88.5%	65.4%
Adult template	0.439 (0.210–0.668)	88.5%	53.8%
All seizure-free patients (*n* = 40)			
PAPT template	0.743 (0.586–0.900)	95.0%	80.0%
Linear template	0.536 (0.362–0.710)	90.0%	62.5%
Adult template	0.469 (0.285–0.653)	90.0%	55.0%

*CI*, confidence interval; *PAPT*, pediatric age–specific PET template

**Fig. 6:**
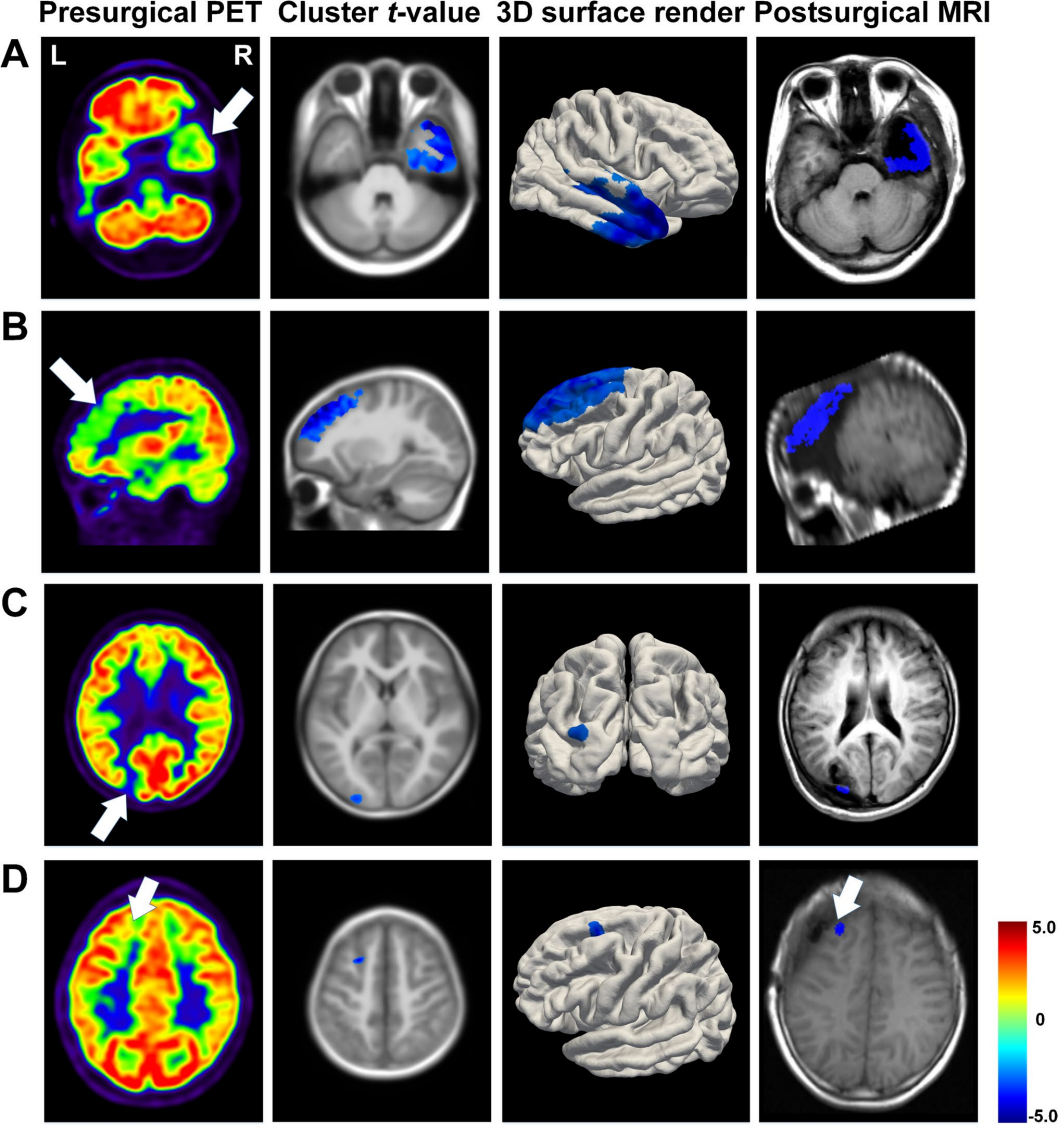
Localization results of patients who underwent epilepsy surgery. **A** Seizure-free 17-year-old girl who underwent right temporal lobe resection, peak-*t* =  − 5.43, cluster size = 30,951; **B** seizure-free 13-year-old girl who underwent left frontal lobe resection, peak-*t* =  − 6.07, cluster size = 29,482; **C** seizure-free 10-year-old boy who underwent left occipital lobe resection, peak-*t* =  − 3.05, cluster size = 174; **D** non-seizure-free 10-year-old boy who underwent left frontal lobe resection. Localization cluster was nearby but without the resection area, peak-*t* =  − 3.90, cluster size = 319 (*P* < 0.01, cluster size > 100)

The PAPT-based analysis detected epileptogenic foci in 38 of 40 (95.0%) postsurgical seizure-free patients, and the overall accuracy was 80.0% (32/40) (Table 2). Figure 6A–C illustrate localization results of three representative seizure-free patients who underwent right temporal lobe, left frontal lobe, and left occipital lobe resection, respectively. For the two patients with negative findings, clusters could be found within resection areas by relaxing *P*-value threshold to 0.05 (Supplementary Fig. 3A–3B). Six patients had confounding clusters, two in the contralateral lobe and four in other lobes (Table 3). In these patients, four clusters within resection areas had the largest sizes.

**Table 3:** Comparison of clusters within resection area and confounding clusters in postsurgical seizure-free patients

Patient	Cluster within resection area			Confounding cluster		
	Localization	Peak-*t*	Size	Localization	Peak-*t*	Size
EP-4	Left temporal lobe	− 3.71	1568*	Left frontal lobe	− 4.49	1391
EP-44	Right frontal lobe	− 4.30	1388	Right temporal lobe	− 6.35	24,696*
EP-65	Left temporal lobe	− 5.85	20,558*	Right temporal lobe	− 6.28	3816
EP-82	Left temporal lobe	− 2.27	252*	Right frontal lobe	− 3.28	146
EP-105	Left frontal lobe	− 3.41	342	Right frontal lobe	− 3.51	226
EP-109	Left frontal lobe	− 3.12	472*	Left temporal lobe	− 3.60	181

^*^Largest cluster size

Metabolic abnormalities were found in 9 of 10 postsurgical non-seizure-free patients (6 girls and 4 boys, mean age = 13.56 years). The patient with negative findings showed cluster within resection area by relaxing *P*-value threshold to 0.05 (Supplementary Fig. 3C). The most significant clusters of two patients were located within the resection areas. One patient showed the most significant cluster nearby but without the resection area (Fig. 6D). Clusters extended to extra-temporal region in two patients with TLE, and four patients showed the most significant clusters in other lobes (Supplementary Table 2).

## Discussion

In this study, we developed a novel age-specific ^18^F-FDG PET template to improve diagnosis of pediatric PET imaging and evaluate its efficiency in pediatric epilepsy. The PAPT we developed significantly improved spatial normalization and dramatically reduced regions with potential registration bias to less than 2% of whole brain volume. As a result, the nearly unbiased spatial normalization essentially improved epileptogenic focus localization. To the best of our knowledge, the PAPT we developed is the first pediatric age–specific PET template created by nonlinear methods.

Spatial normalization is usually a mandatory prerequisite for investigation of PET imaging, which transformed individual PET images into a template space for anatomical correspondences across subjects. However, only very few PET studies reported application of in-house pediatric PET templates, and most creation procedures were not well described for reproducible researches [7, 19, 20]. To our knowledge, only one previous study described detailed template creation based on linear registration between PET images and T1-weighted MRI template [13]. Since the linear registration transforms all voxels in the same manner, misalignment is inevitable in the wrinkled cortical structures of gyri and sulci, which can lead to image blurring in cortex of linear template [12]. In this study, logarithm Jacobian determinant test showed widespread potential registration biases in cortical regions of the linear template, while a rare bias in our PAPT was created by nonlinear methods. Moreover, age ranges of the previous linear template and its dependent MRI template were not well matched. As a result, unwanted global stretching was required to align small pediatric brains to relatively larger age-unspecific linear template, which may introduce systematic biases into diagnosis [21]. Furthermore, our data showed that registration similarity was significantly increased in spatial normalization to the PAPT than those to the linear template and standard adult template. Benefiting from nonlinear registration and iterative optimization methods, the PAPT we developed can nearly eliminate spatial normalization biases and thus provide an optimized anatomical correspondence across subjects, which is essential for subsequent diagnosis and analysis.

Efficiency of our PAPT in clinical diagnosis was evaluated in pediatric epilepsy, which is the most common neurological disorders in children [22]. It is worth noting that routine visual assessment of PET imaging heavily depends on the experience of observers and can miss subtle epileptogenic foci, especially in the extra-temporal regions [23, 24]. To remove the user-dependent biases, voxel-wise statistical methods, such as SPM and Neurostat, have been applied to localization of epileptogenic foci [23–26]. However, the most commonly used PET template provided with SPM was built by averaging and smoothing ^15^O-H_2_O PET images from 12 healthy adults. Due to the large difference between intensity distributions between ^15^O-H_2_O and ^18^F-FDG PET images, the standardized spatial normalization to SPM template could lead to large mismatches [27]. A previous study created ^18^F-FDG PET template by averaging PET images from 17 healthy adults and showed increased sensitivity in group comparison among healthy controls, recent onset, and chronic patients with schizophrenia [8]. A dementia-specific 18F-FDG PET template was also built by averaging PET images from 60 healthy elder healthy and 60 patients with dementia [9] and has been considered the optimized procedure for single-subject dementia analysis [28, 29]. However, these adult templates could not be appropriate for pediatric PET analysis due to the large size and metabolism differences between adults and children [6]. To facilitate pediatric ^18^F-FDG PET analysis, this study developed age-specific ^18^F-FDG PET templates for two age ranges of 6–10 years and 11–18 years, respectively. The human brain reaches 95% of its maximum by the age of 6 years [30], and its glucose metabolic rate stays elevated twice of the adult level between 5 and 10 years [31]. The cerebral metabolic rate slightly declines to adult level during the second decade and shows an inverted U-shaped SUVR curve on ^18^F-FDG PET imaging, whose peak happens around 10 years [32]. Therefore, a cutoff of 10 years was used in this study. For future studies of different age ranges or PET tracers, a new PAPT template can be developed by using the proposed iterative nonlinear registration-averaging optimization approach.

Spatial smoothing can increase signal-to-noise ratio and compensate for inter-subject variations of anatomy. The Gaussian smoothing kernel is recommended at least two to three times the voxel size in single-subject analysis [33]; therefore, our study used a small smoothing kernel with FWHM = 4 mm. Previous studies employed larger smoothing kernels of FWHM = 8–18.6 mm possibly due to the large voxel sizes (2.6–5.5 mm) [23–25, 34, 35]. A larger smoothing kernel can reduce inter-subject voxel-wise variances (Supplementary Fig. 6), which can increase *t*-values in statistical inference. By contrast, the larger smoothing kernel can also draw foci metabolism near to surrounding normal uptake values, leading to reduced *t*-values. The opposed effects can result in complicated changes of peak-*t*-values and cluster extends (Supplementary Fig. 7). In summary, most TLE showed increased absolute peak-*t*-values and cluster sizes, while extra-TLE usually showed reduced absolute peak-*t*-values. The reduced peak-*t*-values could cause negative foci in analyses with FWHM = 8 mm and 12 mm, leading to a reduced sensitivity (Supplementary Table 2). This finding proved the assumption that larger smoothing kernels could reduce sensitivity in previous studies [13, 24]. Because diagnosis of extra-TLE is more challenging than that of TLE in clinical practice [23, 36, 37], a small Gaussian kernel of FWHM = 4 mm could be more appropriate for PET data in our study.

Same to previous epilepsy studies, gender and age were not included as covariates in this study [13, 20, 23–25, 35, 38]. The effect of age could be minimized due to comparison with age-specific controls, and thus, age was not included as a covariate. Moreover, previous studies have shown no significant effect of gender on SUVRs of the most cortical regions [32, 39, 40]; therefore, gender was not included as a covariate. Supplementary Table 3 compares localization with and without covariates, and no significant differences were found by McNemar test (*P* = 1.0). In particular, localization that included age and gender as covariates missed foci in 4 patients, but also recognized foci in another 4 patients. The sizes of all these clusters were near to 100 and could be recognized by a liberal cluster size threshold of *K* > 50. In this study, 19 of 130 (14.6%) patients with epilepsy showed hyper-metabolism on ^18^F-FDG PET images. Although hypo-metabolism is frequently observed on interictal ^18^F-FDG PET images, interictal hyper-metabolism can also be observed in some patients with epilepsy [20, 41–46]. Previous studies showed that hyper-metabolism was associated with high spike frequency on EEG in the absence of ictal events [43, 44]. The interictal hyper-metabolism could reflect increased metabolic activity due to post-seizure restoration of resting membrane poetical and chemical equilibrium, or increased blood–brain barrier permeability for glucose transporters [46]. Still, although parents of the patients and our nuclear medicine staff reported no seizures during ^18^F-FDG uptake and PET scanning, subclinical uptake may not be fully excluded. This limitation could be overcome by a future simultaneous PET-EEG examination.

The proposed PET localization approach did not take into consideration the individual hypothesis on epileptogenic focus from semiology evaluation and EEG examination and thus could form a limitation. A combination of visual assessment and automated approach can essentially improve the epileptogenic focus localization, as described in our previous study [20]. In this study, the PAPT-based approach detected foci in 82.2% of patients with negative visual findings (*n* = 45), and visual assessment could identify 50% of patients with negative findings in PAPT-based analysis. It indicates that visual assessment and SPM analysis can be complementary in epileptogenic focus localization. Moreover, high specificity of the proposed PAPT-based analysis may also improve interpretation of SPM analysis in clinical practice, especially when interpreting multiple clusters within the same lobe (Supplementary Fig. 10). For clinical practice, we suggested a large threshold of *P*-value and cluster size for high sensitivity to epileptogenic focus. Then, visual assessment could exclude false positives and/or include false negatives by considering seizure semiology and EEG findings and concentrate on one or several foci. At last, a restrictive *P*-value threshold could be used to shrink cluster to appropriate sizes for interpretation.

## Conclusion

This study developed a pediatric age–specific ^18^F-FDG PET template by nonlinear approach, which can significantly improve spatial normalization and nearly eliminate registration biases. The unbiased spatial normalization by our PAPT can significantly improve epileptogenic focus localization in pediatric epilepsy, and can also be applied as an important approach to precision diagnosis in pediatric neurological disorders.

## Supplementary Information

Below is the link to the electronic supplementary material.

Supplementary file1 (DOCX 7885 KB)

## Data Availability

The proposed pediatric age–specific ^18^F-FDG PET templates are available from the corresponding authors (Prof. Hong Zhang & Prof. Mei Tian) on reasonable request.
