# Supplementary material for: High-resolution pediatric age–specific 18F-FDG PET template: a pilot study in epileptogenic focus localization
Source: Eur J Nucl Med Mol Imaging. 2021 Nov 8;49(5):1560–73. doi: 10.1007/s00259-021-05611-w (PMC8940757; doi:10.1007/s00259-021-05611-w)
Supplement: Supplementary file 1 — Supplementary file1 (DOCX 7885 KB) [file 259_2021_5611_MOESM1_ESM.docx]

**SUPPLEMENTARY MATERIAL**

**S1. Brain parcellation for pediatric ^18^F-FDG PET template**

Thirty children with epilepsy with centro-temporal spikes (ECTS) who underwent ^18^F-FDG PET/CT and MRI examinations were included to create brain parcellation (14 girls and 16 boys, mean age = 9.83 y). The inclusion criteria were as follows: (1) age between 6 and 18 y; (2) ^18^F-FDG PET/CT and MRI examinations within 1 month; (3) no structural abnormalities on MRI; (4) interval time between last seizure and PET examination > 48 h. All the children had no history of any neuropsychiatric disorders, and no contraindications for MRI.

T1-weighted MRI images were acquired using a 3T MRI scanner with an 8-channel head coil (GE Medical Systems, Signa HDX; USA). A 3D Fast Spoiled Gradient Recalled (3D-FSPGR) sequence was used with following parameters: repetition time (TR) = 7 ms, echo time (TE) = 2.85 ms, flip angle = 8 degree, 150 sagittal slices, field of view (FOV) = 256 × 256 mm^2^, voxel size = 1 × 1 × 1 mm^3^. ^18^F-FDG PET images were acquired by a PET/CT scanner (Biograph mCT; Siemens Medical Solution). All children fasted for at least 6 h, and withdrew anti-epileptic drugs overnight (> 12 h) before PET examination. Children were injected with a standard dose of ^18^F-FDG (3.7 MBq/kg), rested in a dark quiet environment for 40 mins, and then underwent a 5-minute PET/CT examination.

FreeSurfer software (<https://surfer.nmr.mgh.harvard.edu>) was used to preprocess T1-weighted MRI images. The preprocessing steps included skull stripping, bias field correction, gray-white matter segmentation, cortical surface reconstruction, cortex and subcortical structure segmentation. The preprocessed T1-weighted images were then coregistered with corresponding PET images by rigid registration using a boundary-based cost function. Parcellation according to Desikan-Killiany atlas was also transformed to the individual PET space by the rigid transformation. ^18^F-FDG PET images were spatially normalized to unbiased age-specific ^18^F-FDG PET templates, followed by warping Desikan-Killiany parcellation using nearest-neighbor interpolation. A Gaussian kernel with 4 mm full width half maximum (FWHM) was used to create brain parcellation for the pediatric age-specific ^18^F-FDG PET templates. For each voxel within the template space, the Gaussian kernel was applied. For each voxel of the template space, summed probabilities of parcellation ROIs within the Gaussian kernel were determined by iterating all subjects. The voxel was assigned to the parcellation ROI with the maximum summed probability. Supplementary Fig. 1 showed the brain parcellation for pediatric age-specific ^18^F-FDG PET template.


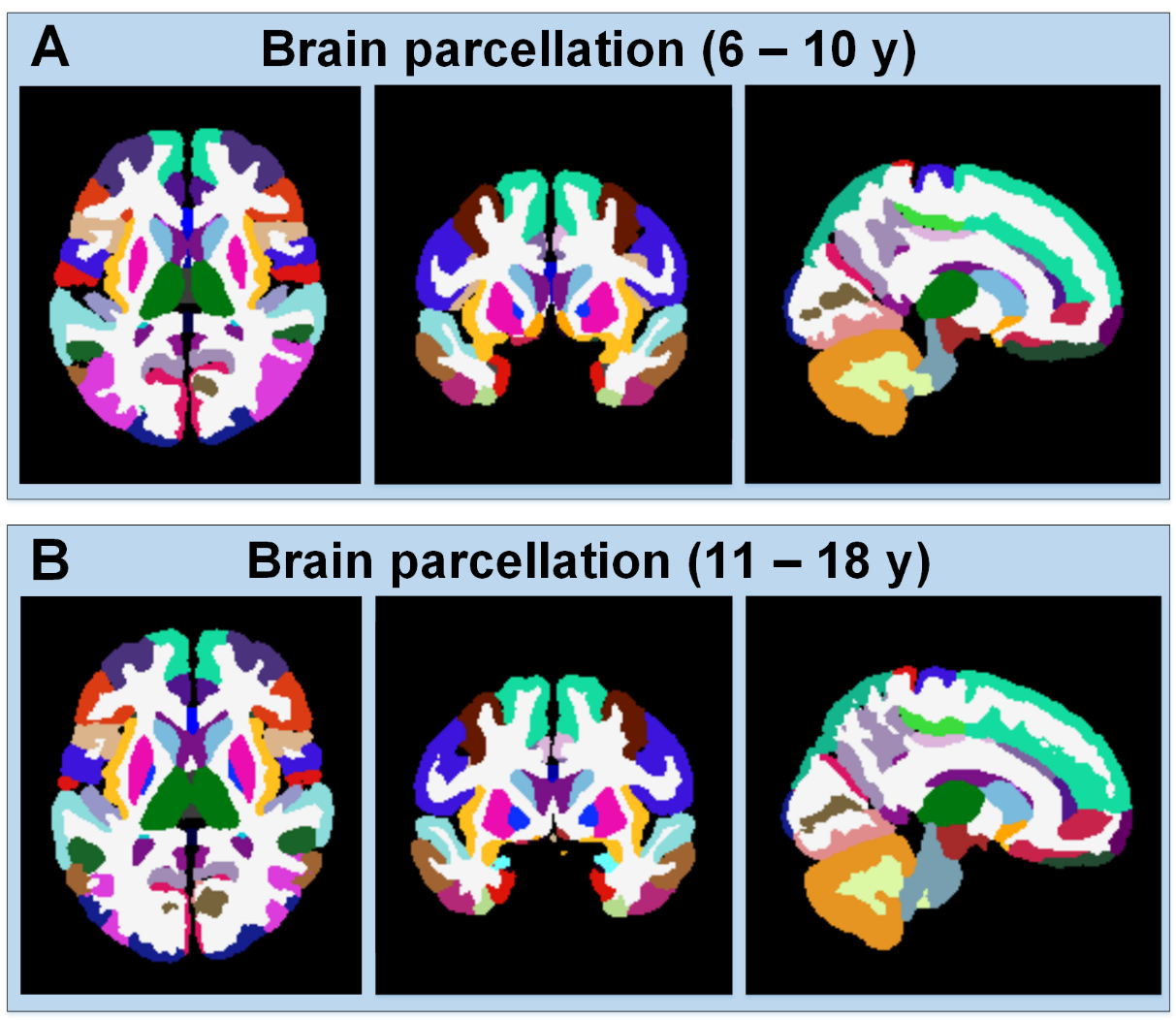


**Supplementary Fig. 1.**  Brain parcellation for the pediatric age-specific ^18^F-FDG PET templates. The parcellation was created according to Desikan-Killiany atlas for two age ranges (A) 6 – 10 y and (B) 11 – 18 y. The brain parcellation provides brain partition information, and can be used in future region-of-interest based analysis, network analysis, ect.


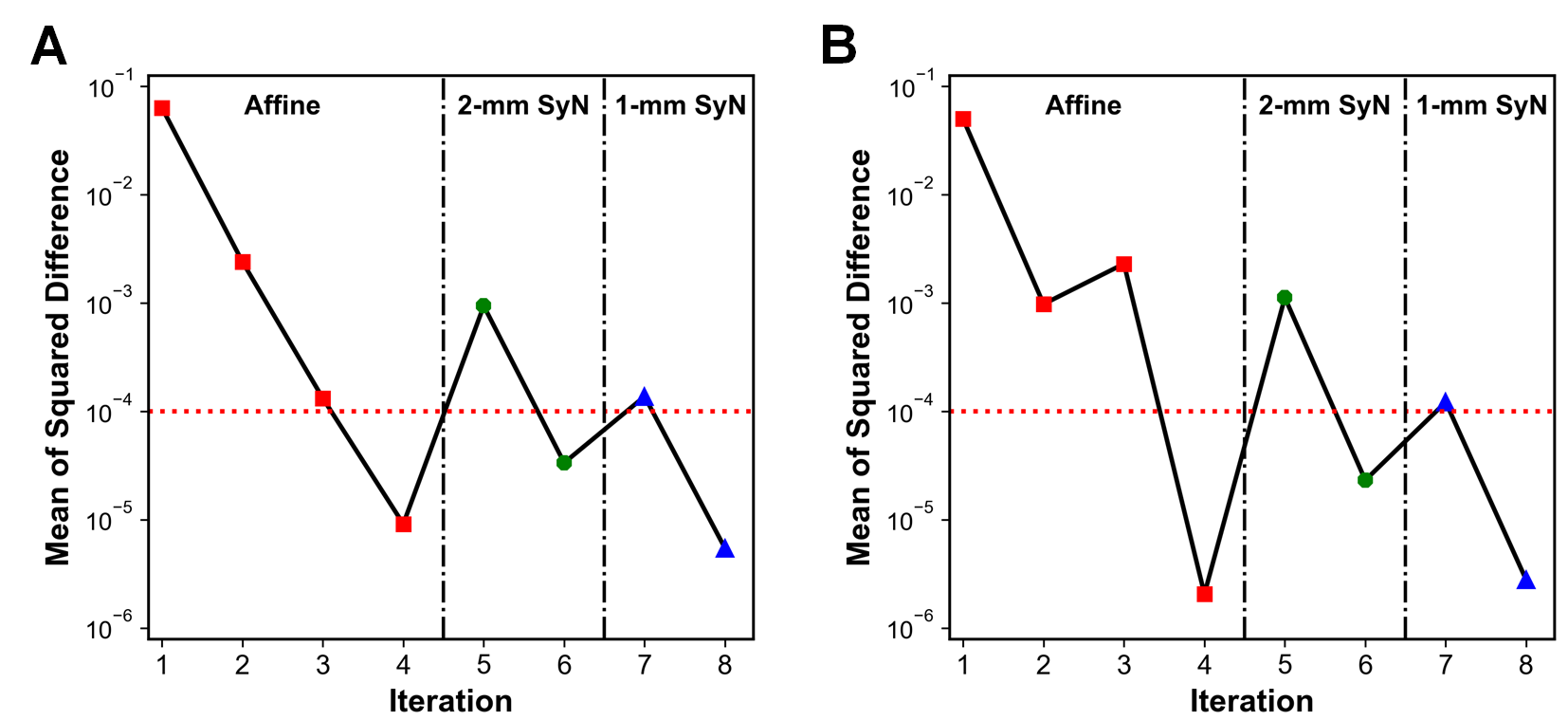


**Supplementary Fig. 2.** Convergence process to develop pediatric age-specific PET template. (A) 6 – 10 y; (B) 11 – 18 y. Both convergence processes took eight iterations, including four affine registration iteration, two 2-mm symmetric diffeomorphic registration normalization (SyN) iterations and two 1-mm SyN iterations.


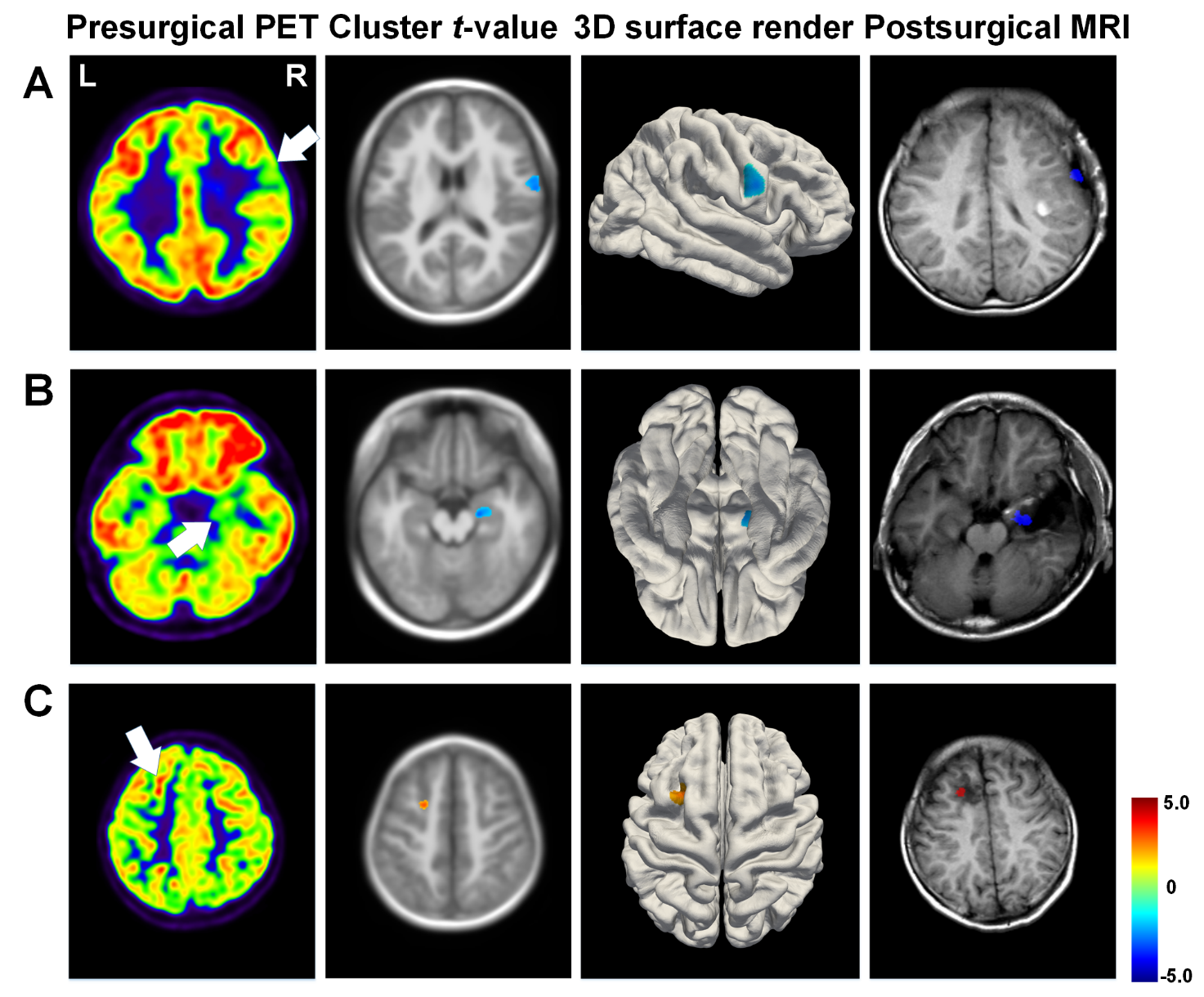


**Supplementary Fig. 3. Revisiting focus localization of postsurgical patients with negative findings.** (A) Seizure-free 11 y girl who underwent right precentral gyrus resection, peak-*t* = -2.68, cluster size = 967. (B) Seizure-free 14 y boy who underwent right temporal-hippocampus-amygdala resection, peak-*t* = -2.78, cluster size = 463. (C) Non-seizure-free 10 y girl who underwent left caudal middle frontal lobe resection, peak-*t* = 2.99, cluster size = 184 (*P* < 0.05, cluster size > 100).

**Supplementary Table 1.** Logarithm global scaling to align validation PET images.

|  |  | **Direction** | **Log-scaling** | **P-value** | **95% CI** |
| --- | --- | --- | --- | --- | --- |
|  | |  |  |  |  |
| 6 – 10 y group (n = 16) | |  |  |  |  |
|  | PAPT template | L-R | -0.011 ± 0.033 | 0.220 | -0.028 – 0.007 |
|  |  | A-P | -0.014 ± 0.038 | 0.168 | -0.034 – 0.007 |
|  |  | I-S | -0.012 ± 0.029 | 0.126 | -0.027 – 0.004 |
|  | Linear template | L-R | 0.055 ± 0.033 | < 0.001 | 0.037 - 0.072 |
|  |  | A-P | 0.136 ± 0.038 | < 0.001 | 0.115 – 0.156 |
|  |  | I-S | 0.167 ± 0.029 | < 0.001 | 0.152 – 0.183 |
|  | Adult template | L-R | 0.066 ± 0.034 | < 0.001 | 0.048 – 0.084 |
|  |  | A-P | 0.120 ± 0.039 | < 0.001 | 0.099 – 0.141 |
|  |  | I-S | 0.200 ± 0.031 | < 0.001 | 0.183 – 0.216 |
|  | |  |  |  |  |
| 11 – 18 y group (n = 19) | |  |  |  |  |
|  | PAPT template | L-R | 0.003 ± 0.045 | 0.754 | -0.018 – 0.025 |
|  |  | A-P | 0.006 ± 0.041 | 0.507 | -0.013 – 0.026 |
|  |  | I-S | -0.003 ± 0.030 | 0.682 | -0.017 – 0.012 |
|  | Linear template | L-R | 0.028 ± 0.045 | 0.015 | 0.006 – 0.050 |
|  |  | A-P | 0.091 ± 0.041 | < 0.001 | 0.071 – 0.110 |
|  |  | I-S | 0.121 ± 0.030 | < 0.001 | 0.107 – 0.136 |
|  | Adult template | L-R | 0.060 ± 0.047 | < 0.001 | 0.037 – 0.082 |
|  |  | A-P | 0.101 ± 0.482 | < 0.001 | 0.081 – 0.121 |
|  |  | I-S | 0.194 ± 0.034 | < 0.001 | 0.178 – 0.211 |

CI = confidence interval; L-R = left-right; A-P = anterior-posterior; I-S = inferior-superior.

**Supplementary. Table 2.** Comparison of clusters within and without resection area in postsurgical non-seizure-free patients

| Patient | Cluster within resection area | | |  | Cluster without resection area | | |
| --- | --- | --- | --- | --- | --- | --- | --- |
|  | Localization | Peak-t | Size |  | Localization | Peak-t | Size |
|  |  |  |  |  |  |  |  |
| EP-16 | Left temporal lobe | -5.72 | 58123* |  | Right temporal lobe | -5.87 | 40299 |
| EP-64 | Right superior lobe | -2.84 | 163 |  | Left superior lobe | -4.35 | 815* |
| EP-102 | Left frontal lobe | -3.40 | 760 |  | Right temporal lobe | -3.51 | 226 |
| EP-110 | Left temporal lobe | -3.23 | 131 |  | Right temporal lobe | -3.32 | 257 |

* Largest cluster size.

**Supplementary Table 3.** Localization results with including age and sex as covariates.

|  | Kappa (95% CI) | Detection rate | Accuracy |
| --- | --- | --- | --- |
|  |  |  |  |
| 6 – 10 y group (n = 48) | 0.717 (0.576 – 0.857) | 81.3% | 75.0% |
| 11 – 18 y group (n = 82) | 0.755 (0.650 – 0.860) | 93.9% | 80.5% |
| All patients (n = 130) | 0.748 (0.666 – 0.830) | 89.2% | 78.5% |

CI = confidence level.

**S2. Comparison of registration approaches**

In this study, diffeomorphic normalization registration (SyN) registration was used for spatial normalization since it achieved the best performance in comparison of 14 nonlinear registration algorithm by a previous study [1]. We compared spatial normalization results of SyN registration and SPM registration. The SPM parameters were selected according to a previous study [2], and as follows: bounding box = [-90:91; -126:91; -72:109], nonlinear frequency cutoff = 25, iterations = 16, and regularization = 1. Inter-subject similarities among normalized images were compared using Mattes mutual information and normalized cross correlation. As shown in Supplementary Fig. 4, image similarities were significantly improved by using SyN registration compared with SPM registration (*P* < 0.001). Supplementary Fig. 5 demonstrated spatial normalization results of two subjects to age-specific PET template by SPM and SyN registration approaches. Canny edges were extracted and overlaid with the other normalized image to indicate anatomical mismatches. Remarkable mismatches can be found in the normalized images by SPM registration, while Canny edges well matched normalized image of the different subject by SyN registration. Therefore, the SyN registration was used for spatial normalization in this study.


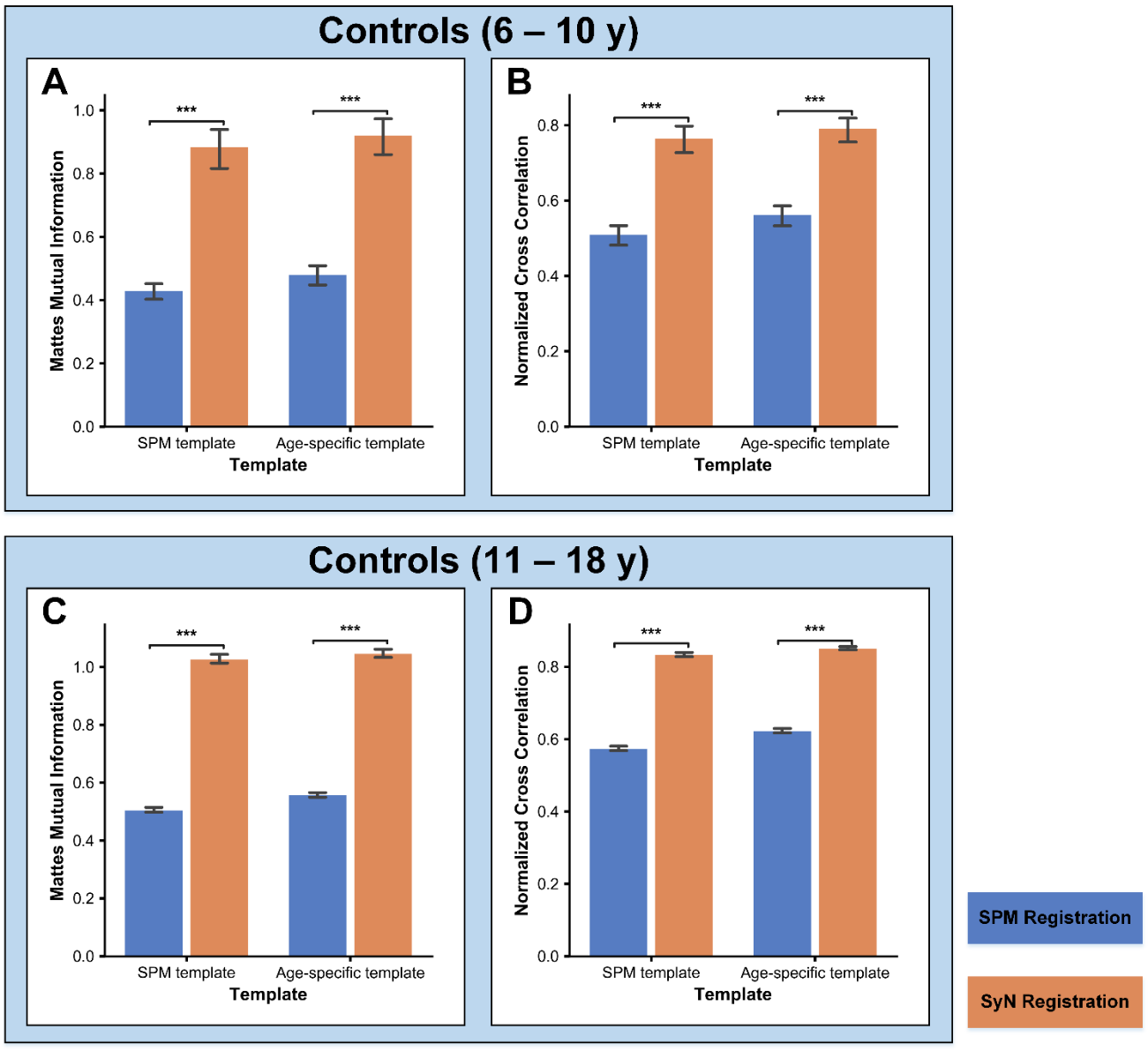


**Supplementary Fig. 4.**  Comparison of inter-subject similarities among normalized images. (A – B) Mattes mutual information and normalized cross correlation in 6 – 10 y group, respectively. (C – D) 11 – 18 y group. The SyN registration significantly improved inter-subject similarities compared with SPM registration.


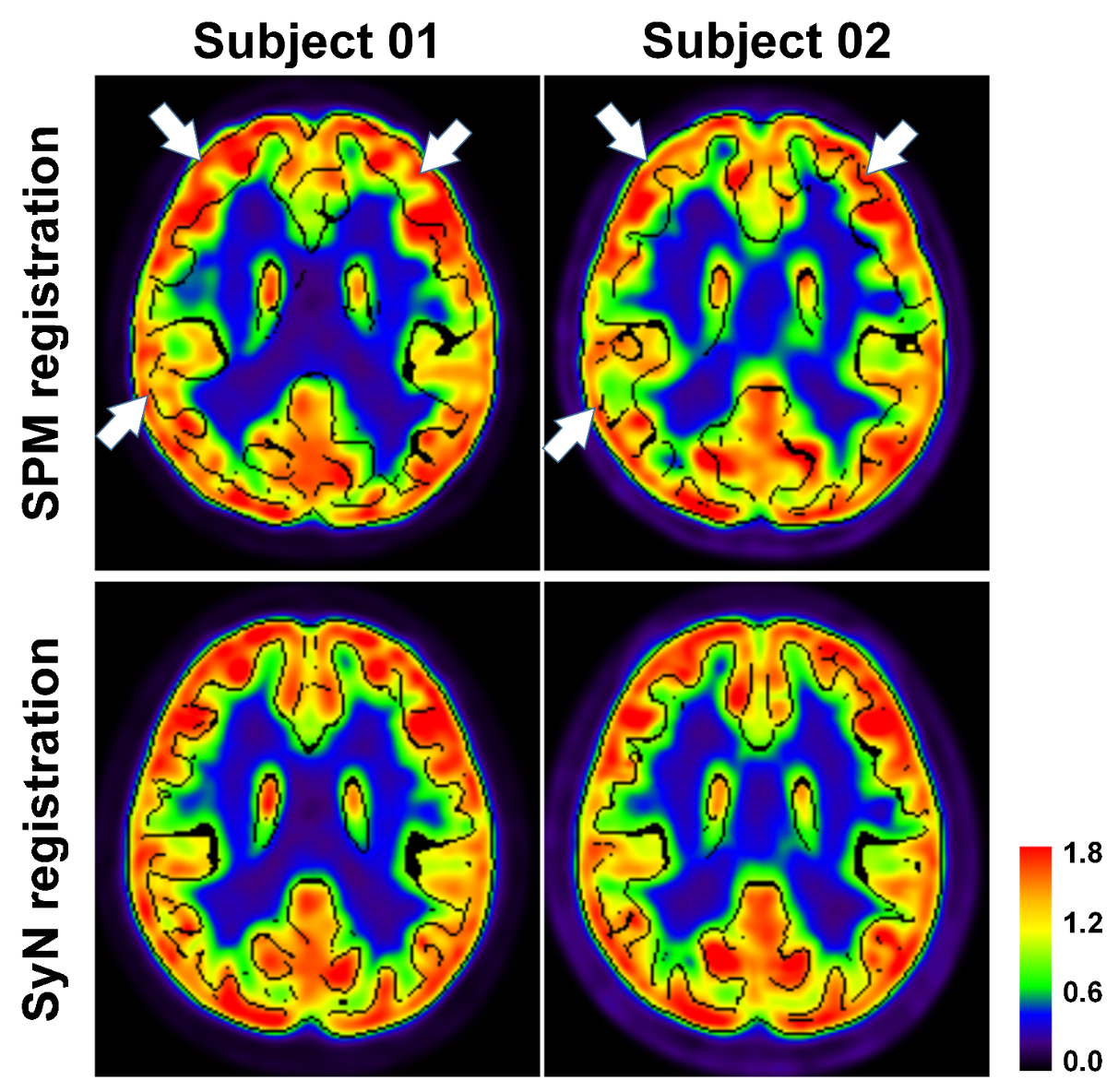


**Supplementary Fig. 5.**  Comparison of spatial normalization results of two subjects. Individual ^18^F-FDG PET images were spatially normalized to pediatric age-specific ^18^F-FDG PET template by SPM or SyN registration approaches. The black contours are Canny edges from the other subject, and white arrows point to anatomical mismatches between two normalized images by SPM registration.

**S3. Influence of Gaussian smoothing kernels**

To investigate influence of smoothing kernels, we re-implemented the localization experiments by using Gaussian smoothing kernels of FWHM = 4 mm, 8 mm, and 12 mm, respectively. As shown in Supplementary Table 4, the detection rates declined for all localization approaches when larger smoothing kernels were employed. Let specificity denote ratio of correctly localized foci to all detected foci, *i.e.* accuracy / detection rate). The linear template-based analysis showed increased specificities from 79.2% to 85.4% and 87.8% by using larger smoothing kernels, while PAPT and adult template based analyses had robust specificities of about 90% and 70%, respectively. Considering that the sensitivity (detection rate) is more important than specificity in clinical practice, the smoothing kernel of FWHM = 4 mm can be more appropriate for PET scans in this study.

A larger smoothing kernel can reduce intra-subject voxel-wise variance of SUVRs, especially at the peak voxels. For the PAPT-based analysis in 11 – 18 y children, the maximal variances of controls were 0.44, 0.28 and 0.20 for FWHM = 4 mm, 8 mm and 12 mm, respectively. Because SPM modifies variances by adding 1/1000 of the maximum to avoid problems of very low variance, shrinkage of variances can be amplified for large smoothing kernels, as demonstrated in Supplementary Fig. 6. The larger smoothing kernel could bring smaller intra-subject variance, which can **increase *t*-values** of statistical analysis. By contrast, the larger smoothing kernel could also exacerbate partial volume effect, and draw foci metabolism near to surrounding normal uptake levels, resulting in **reduced *t*-values**.

The opposed effects can lead to complicated changes of peak-*t* values and cluster extents. For example, when a larger Gaussian smoothing kernel was used, the absolute peak-*t* value and cluster size could increase (Supplementary Fig. 7A) or decrease (Supplementary Fig. 7B), or the absolute peak-*t* value decreased but cluster size increased (Supplementary Fig. 7C). It is noteworthy that most temporal lobe epilepsy (TLE) showed increased absolute peak-*t* values and cluster sizes when employing a large smoothing kernel, while the extra-temporal lobe epilepsy (extra-TLE) usually showed decreased absolute peak-*t* values. All the disappeared foci using large smoothing kernels belonged to extra-TLE, which could be caused by the reduced absolute peak-*t* values and cluster sizes. Because diagnosis of extra-TLE is more challenging than that of TLE in clinical practice [3–5], it is suggested to employ a small Gaussian kernel for the PAPT-based analysis.

**Supplementary Table 4.** Comparison of focus localization results by using different smoothing kernels.

|  | FWHM = 4 mm | |  | FWHM = 8 mm | |  | FWHM = 12 mm | |
| --- | --- | --- | --- | --- | --- | --- | --- | --- |
|  | Detection rate | Accuracy |  | Detection rate | Accuracy |  | Detection rate | Accuracy |
|  |  |  |  |  |  |  |  |  |
| PAPT template | 89.2% | 80.0% |  | 87.7% | 78.5% |  | 85.4% | 76.9% |
| Linear template | 81.5% | 64.6% |  | 79.2% | 67.7% |  | 75.4% | 66.2% |
| Adult template | 80.8% | 58.5% |  | 80.8% | 56.2% |  | 80.0% | 58.5% |

FWHM = full-width half maximum


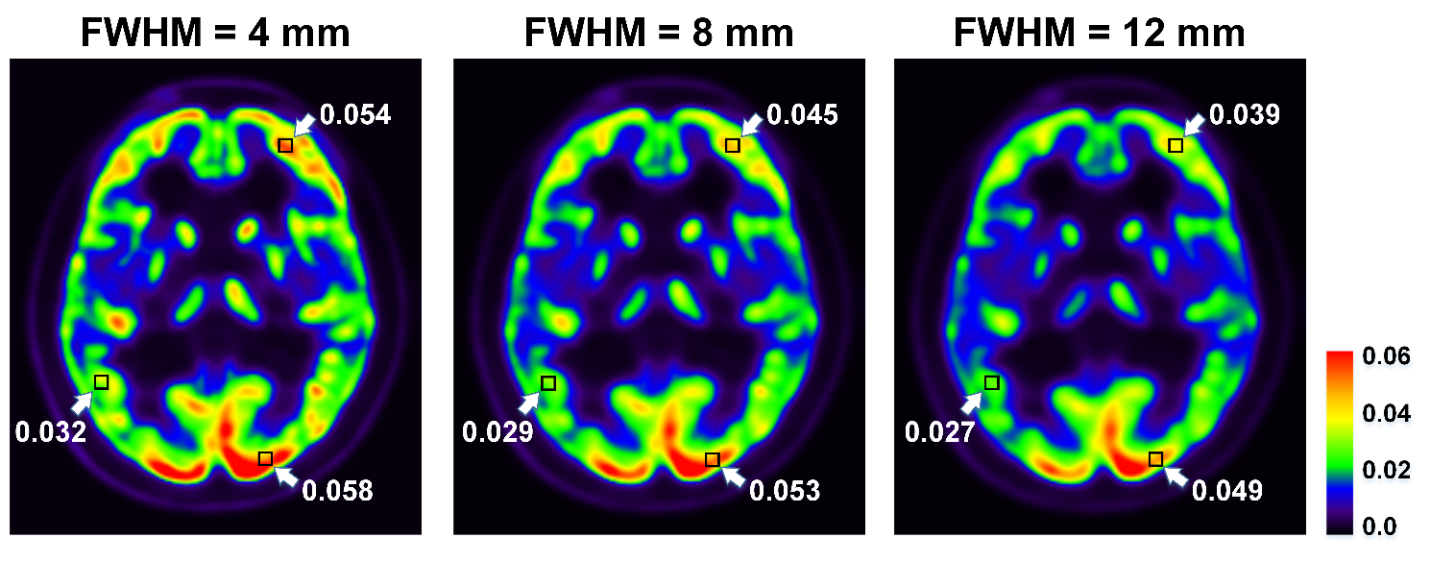


**Supplementary Fig. 6.** Variance images with respect to Gaussian smoothing kernels with full-width half maximum (FWHM) = 4 mm, 8mm, and 12 mm, respectively. The larger FWHM, the smaller intra-subject variance.

**
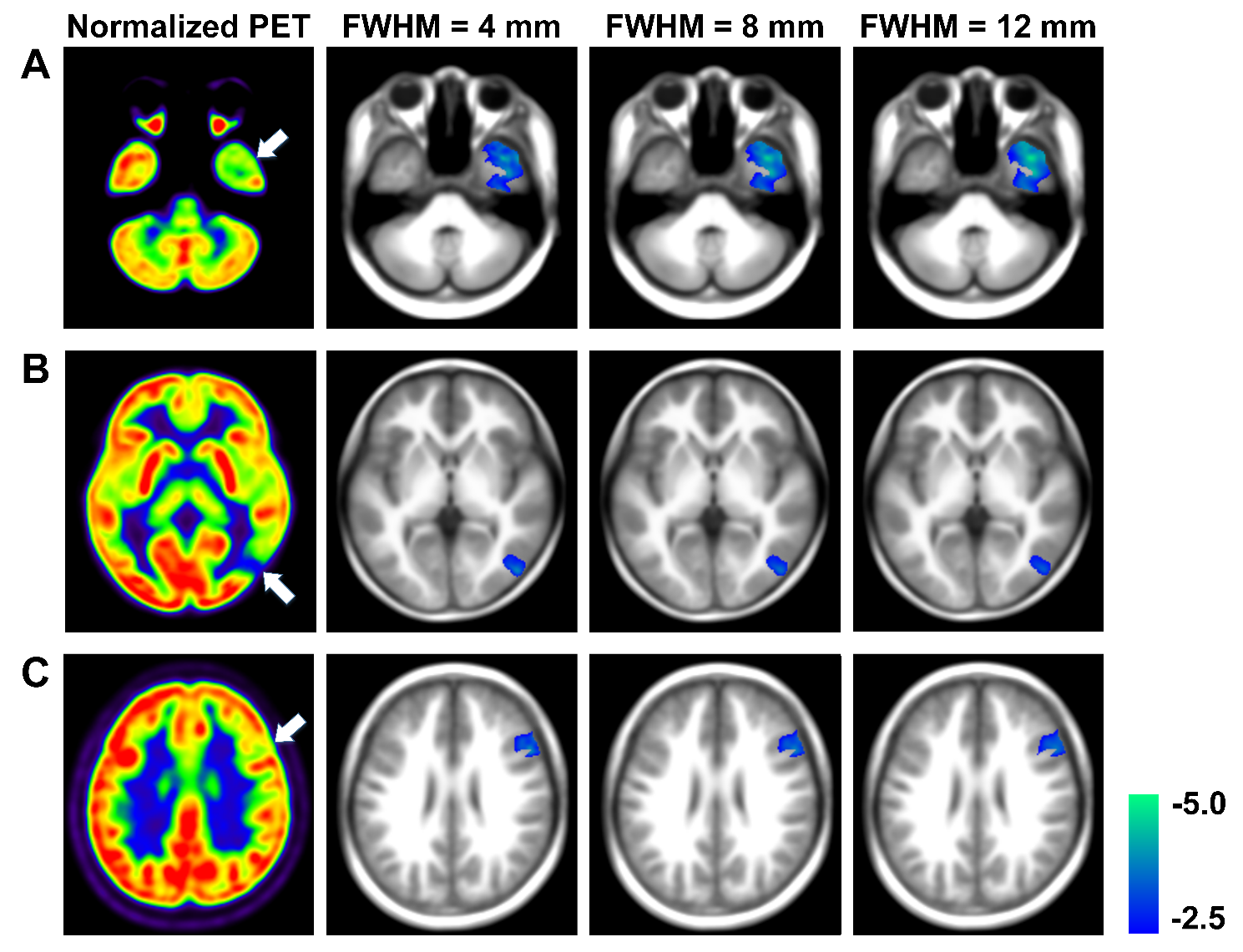
**

**Supplementary Fig. 7.** Localization results by using different Gaussian smoothing kernels. When a larger Gaussian kernel is used, (A) peak-*t* values (-4.36, -4.72, and -5.05) and cluster sizes (21394, 25507, and 28526) increased; (B) peak-*t* values (-3.80, -3.62, and -3.44) and cluster size (830, 747, and 671) decreased; (C) peak-*t* values (-4.64, -4.46, and -4.29) decreased and cluster size (1480, 1614, and 1745) increased.

**S4. *P*-value and cluster size thresholds in focus localization**

We evaluated influence of P-value and cluster sizes on focus localization since these patients had surgical outputs as gold standard. The focus cluster sizes were evaluated by using thresholds of uncorrected *P*-value < 0.001, 0.002, 0.005, 0.01, 0.02, and 0.05, respectively. The corrected *P*-value < 0.05 was not used because this threshold was too restrictive for single-subject SPM analysis, and could reduce sensitivity of focus detection [3,6]. As shown in Supplementary Fig. 8, the cluster sizes jumped between *P*-value thresholds of 0.005 and 0.02, and all focus clusters reached 100 continuous voxels at *P*-value < 0.02. Supplementary Fig. 9 demonstrated focus clusters of two representative patients by using different *P*-value thresholds. In Supplementary Fig. 9A, no voxels reached significance level of 0.001 and 0.002. The focus clusters sizes were 10, 252, and 6392 by using thresholds of *P* < 0.005, 0.01, and 0.05, respectively. In Supplementary Fig. 9B, the focus cluster sizes were 783, 3469, and 26845 by using thresholds of P < 0.001, 0.01, and 0.05, respectively. The blue cluster (P < 0.05) extended outside resection area from parietal lobe (left supramarginal gyrus) to frontal lobe (left caudal middle frontal lobe and precentral gyrus).

Supplementary Table 5 and 6 compared localization results by using different P-value and cluster size thresholds (K > 50, 100, and 200). A threshold of larger P-value and smaller cluster size could increase sensitivity of focus detection, and reached 100% at P < 0.02 uncorrected and K > 100. However, a liberal P-value threshold could also lead to larger clusters extending to other lobes, as shown in Supplementary Fig. 9B, which may reduce specificity of focus interpretation. Therefore, we used the threshold of P < 0.01 uncorrected and K > 100 to balance sensitivity and specificity.


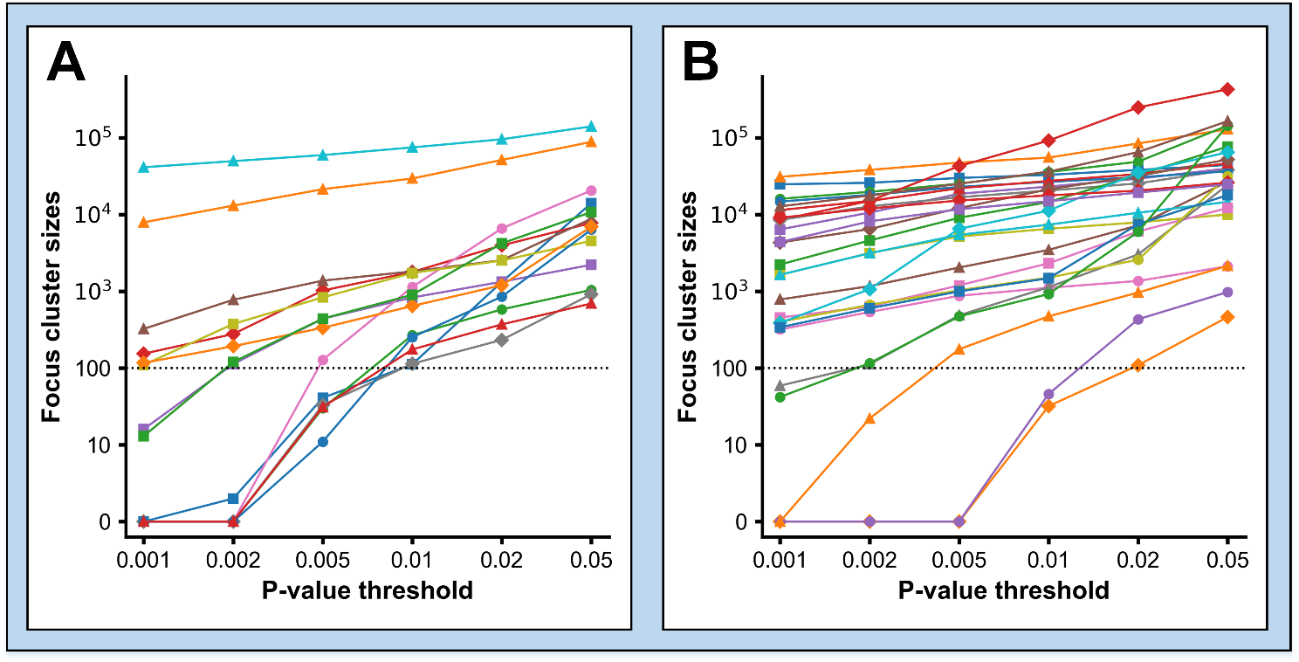


**Supplementary Fig. 8.** Focus cluster sizes of post-surgical seizure-free patients. (A) 6 – 10 y patients; (B) 11 – 18 y patients.


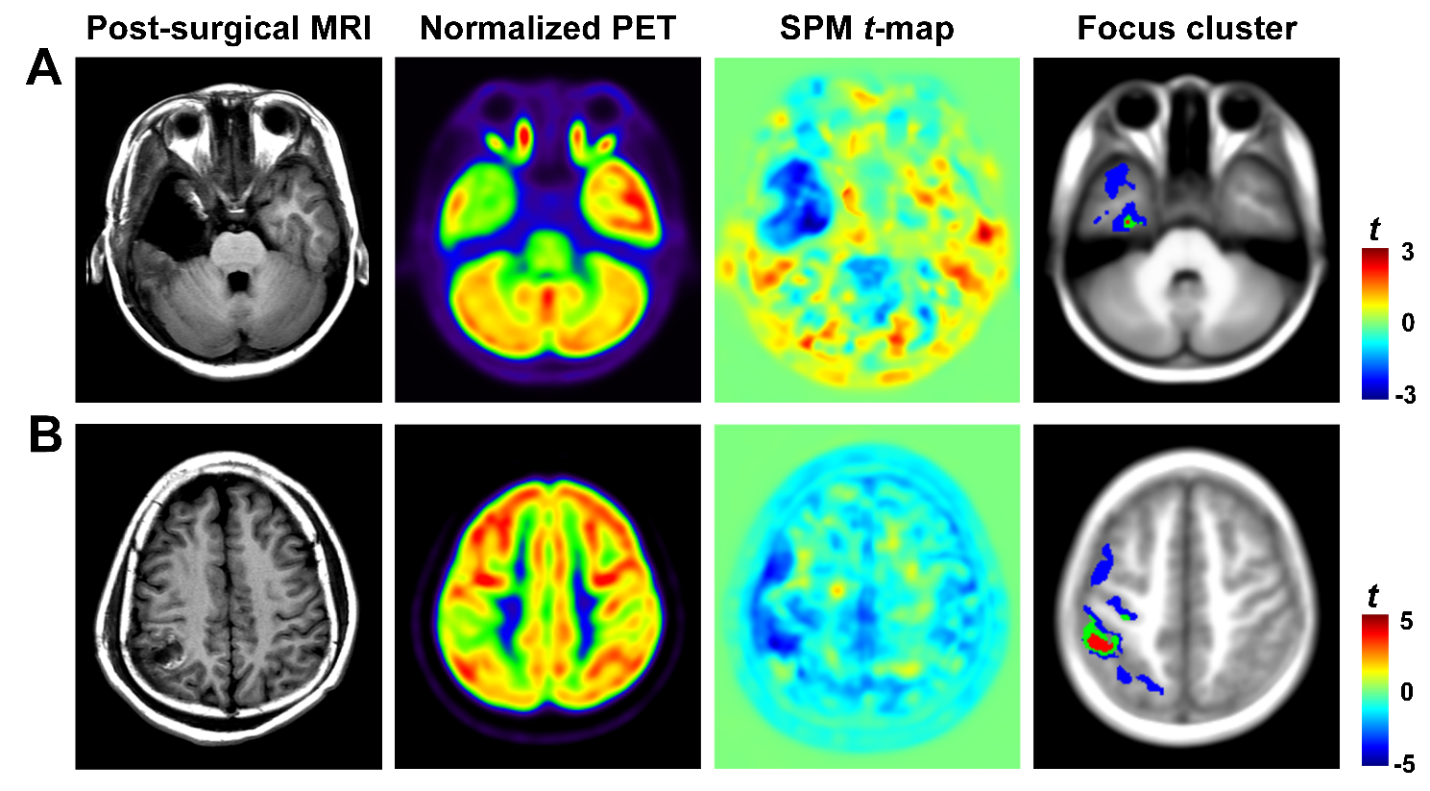


**Supplementary Fig. 9.** Localization results by using different thresholds of *p*-values. (A) An 8-y girl who underwent temporal-lobe epilepsy surgery. The red, green and blue clusters denote localization results by using *p*-value thresholds of 0.005, 0.01, and 0.05, respectively. (B) A 12-y boy who underwent parietal-lobe epilepsy surgery. The red, green and blue clusters denote localization results by using *p*-value thresholds of 0.001, 0.01, and 0.05, respectively.

**Supplementary Table 5.** Localization results of post-surgical seizure-free 6 – 10 y patients (n = 14) by using different *P*-value and cluster size thresholds

|  | K > 50 | |  | K > 100 | |  | K > 200 | |
| --- | --- | --- | --- | --- | --- | --- | --- | --- |
|  | Detection rate | Accuracy |  | Detection rate | Accuracy |  | Detection rate | Accuracy |
|  |  |  |  |  |  |  |  |  |
| P < 0.001 | 42.9% | 42.9% |  | 42.9% | 42.9% |  | 21.4% | 21.4% |
| P < 0.002 | 57.1% | 57.1% |  | 57.1% | 57.1% |  | 35.7% | 35.7% |
| P < 0.005 | 64.3% | 64.3% |  | 64.3% | 64.3% |  | 57.1% | 57.1% |
| P < 0.01 | 100% | 85.7% |  | 100% | 85.7% |  | 78.6% | 71.4% |
| P < 0.02 | 100% | 85.7% |  | 100% | 85.7% |  | 100% | 85.7% |
| P < 0.05 | 100% | 85.7% |  | 100% | 85.7% |  | 100% | 85.7% |

**Supplementary Table 6.** Localization results of post-surgical seizure-free 11 – 18 y patients (n = 26) using different *P*-value and cluster size thresholds

|  | K > 50 | |  | K > 100 | |  | K > 200 | |
| --- | --- | --- | --- | --- | --- | --- | --- | --- |
|  | Detection rate | Accuracy |  | Detection rate | Accuracy |  | Detection rate | Accuracy |
|  |  |  |  |  |  |  |  |  |
| P < 0.001 | 84.6% | 73.1% |  | 80.8% | 69.2% |  | 80.8% | 69.2% |
| P < 0.002 | 88.5% | 76.9% |  | 88.5% | 76.9% |  | 80.8% | 69.2% |
| P < 0.005 | 92.3% | 76.9% |  | 92.3% | 76.9% |  | 88.5% | 76.9% |
| P < 0.01 | 92.3% | 76.9% |  | 92.3% | 76.9% |  | 92.3% | 80.8% |
| P < 0.02 | 100% | 80.8% |  | 100% | 80.8% |  | 96.2% | 80.8% |
| P < 0.05 | 100% | 80.8% |  | 100% | 80.8% |  | 100% | 80.8% |


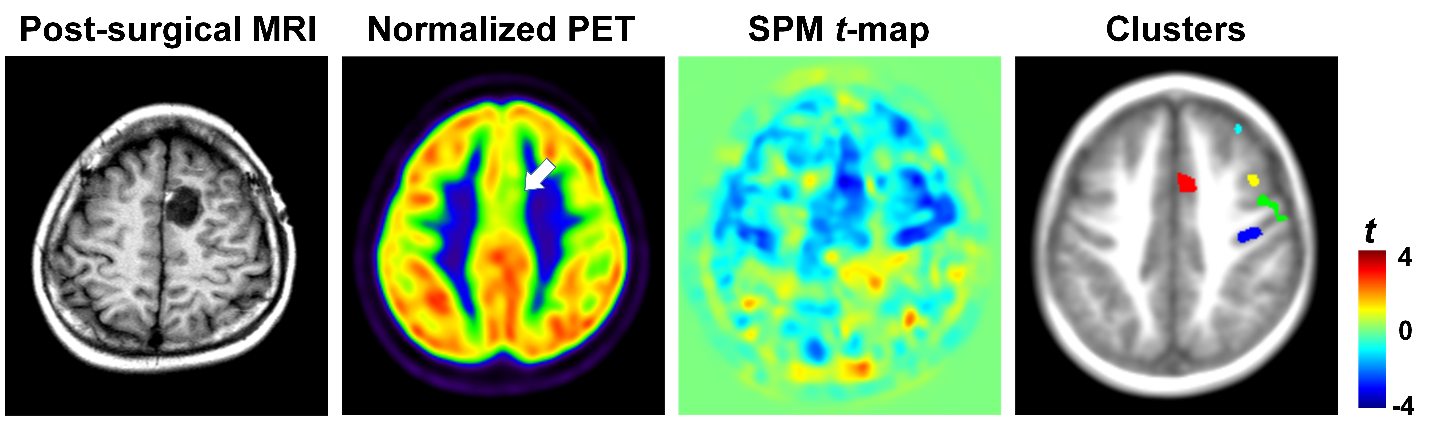


**Supplementary Fig. 10.** Localization result of a post-surgical seizure-free patient with multiple foci in the same lobe. The red focus had the highest absolute peak-*t* value in proposed PAPT-based analysis, while the green focus had the highest values in linear template and adult template-based analyses.

**References**

1. Klein A, Andersson J, Ardekani BA, Ashburner J, Avants B, Chiang MC, et al. Evaluation of 14 nonlinear deformation algorithms applied to human brain MRI registration. Neuroimage. 2009;46:786–802. doi:10.1016/j.neuroimage.2008.12.037.

2. London K, Howman-Giles R. Voxel-based analysis of normal cerebral [18F]FDG uptake during childhood using statistical parametric mapping. Neuroimage. 2015;106:264–71. doi:10.1016/j.neuroimage.2014.11.047.

3. Kim YK, Lee DS, Lee SK, Chung CK, Chung JK, Lee MC. 18F-FDG PET in localization of frontal lobe epilepsy: Comparison of visual and SPM analysis. J Nucl Med. 2002;43:1167–74.

4. Tomás J, Pittau F, Hammers A, Bouvard S, Picard F, Vargas MI, et al. The predictive value of hypometabolism in focal epilepsy: a prospective study in surgical candidates. Eur J Nucl Med Mol Imaging. 2019;46:1806–16. doi:10.1007/s00259-019-04356-x.

5. Rathore C, Dickson JC, Teotónio R, Ell P, Duncan JS. The utility of 18F-fluorodeoxyglucose PET (FDG PET) in epilepsy surgery. Epilepsy Res. 2014;108:1306–14. doi:10.1016/j.eplepsyres.2014.06.012.

6. Mayoral M, Marti-Fuster B, Carreño M, Carrasco JL, Bargalló N, Donaire A, et al. Seizure-onset zone localization by statistical parametric mapping in visually normal 18F-FDG PET studies. Epilepsia. 2016;57:1236–44. doi:10.1111/epi.13427.
